# Mapping the themes and intellectual structure of micro-influencers: A systematic review and meta-analysis

**DOI:** 10.12688/f1000research.175286.1

**Published:** 2026-01-16

**Authors:** L.M.D.O Wimalasena, D.M.R Dissanayake

**Affiliations:** 1 University of Kelaniya Faculty of Commerce and Management Studies, Kelaniya, Western Province, 11600, Sri Lanka; 2 Department of Marketing Management, University of Kelaniya Faculty of Commerce and Management Studies, Kelaniya, Western Province, 11600, Sri Lanka; 3 Adjunct Professor, Taylor’s Business School, Taylor's University, Subang Jaya, Selangor, Malaysia

**Keywords:** Micro influencers, Influencer marketing, Nano influencers, Bibliometric analysis, VOSviewer, Biblioshiny, Systematic Literature Review, Social media

## Abstract

**Background:**

Social media marketing dynamics have been reshaped with the rise of micro-influencers, with the offering of community-driven and authentic brand engagement that goes beyond traditional celebrity endorsements and advertising. This study aims to systematically examine the micro-influencer research published between 2020 and 2025, focusing on identifying themes, research gaps, methodological characteristics, and providing future directions.

**Methods:**

This paper focuses on providing a thorough systematic literature review (SLR) incorporating the PRISMA protocol on micro-influencers, while identifying interdependencies and relationships. 68 research papers that were derived from the Scopus database were analysed through bibliometric and content analysis. Bibliometric analysis was conducted using a combination of VOSviewer and Biblioshiny packages. Results were analysed through thematic clusters, co-occurrence, and bibliographic coupling analysis.

**Results:**

The analysis depicts a steady growth in publications accelerated with digitalisation and post-COVID consumer shift that placed a commercial value on small-scale influencers. Research is fragmented across authenticity, trust, engagement, sponsorship disclosure, and niche-domains for micro-influencers. The literature is geographically skewed towards the West and high-income Asian countries. Less longitudinal evidence with strong methodological homogeneity was present. Algorithmic visibility, emerging platform comparisons, influencer labour, and non-commercial uses of micro-influencers were underexplored themes. Five research areas were identified from clustering.

**Conclusion:**

Despite the rapid expansions, micro-influencer literature remains theoretically fragmented and unevenly distributed. This review provides a comprehensive analysis of the literature on micro-influencers and provides implications and future research directions. Most studies focused on analysing a single platform with cross-sectional designs. This study highlights the need for thorough theoretical integration with methodological diversification, considering emerging platforms to better understand how micro influencers shape digital communication.

## Introduction

The wide use of social media in recent years has transformed the marketing and consumer engagement landscape profoundly. Considering all the significant evolutions, the evolution of influencer marketing holds a high position. This strategy involves individuals who possess a significant number of followers to actively engage in promoting brands or products with the intention of receiving monetary compensation or otherwise. ^1^ Social proof theory acts as the basis of this sort of paradigm shift, and this explains the behaviour of people to conform to the actions of someone they trust or highly admire. ^2^ This psychological tendency is capitalized in influencer marketing, which is identified as a powerful tool in brand communication strategies in the digital era. In order to achieve various goals of brands, ranging from increasing awareness to increasing direct sales, currently more than 90% of the brands utilize influencers in their marketing efforts. ^3^ Various types of influencers can be identified based on the number of followers they possess, and a significant shift from reliance on mega influencers with millions of followers to micro-influencers typically having followers between 5000 to 50,000 is evident. ^4^ Micro-influencers are known to be more authentic, community-oriented, and approachable than other influencers, which allows them to form parasocial relationships that are strong in nature with their audience. ^5^

Studies have proven that these relationships result in enhancing perceived credibility, brand engagement, and consumer trust. ^6^ Higher engagement rates achieved by micro-influencers compared to mega influencers have proven the effectiveness of utilizing micro-influencers in community building, niche marketing, and driving purchase decisions. ^7, 8^ Cost-effective campaigns on platforms such as Instagram, YouTube, and TikTok, targeting segmented audiences, utilize them. ^3^ Nano-influencers with less than 5000 followers are also significantly utilized in hyper-targeted marketing efforts. ^9^ Though the reach is comparatively small, high trust and intimate follower engagement are crucial in grassroots influence and brand authenticity. ^10^ Research remains scarce in relation to the effectiveness and strategic roles. In the domain of micro-influencer systematic literature reviews, a dearth of literature exists in peer-reviewed journals. Chen et al., ^11^ which was among the few reviews in this domain, employed CiteSpace while considering literature from 2013 to 2024. Though it covered from 2013, it did not only consider social sciences, limiting coherent theoretical synthesis relevant to advertising, consumer behavior, persuasion, digital labor, and communication scholarship. Our review employed a narrower, contextually relevant snapshot of the domain, which consists of post-COVID era emerging themes with digital acceleration, platform shifts, rapid advancements in artificial intelligence, and the creator economy surge.

A notable gap exists in the lack of studies employing advanced tools such as VOSviewer and Biblioshiny employing PRISMA, to conduct a bibliometric analysis with systematic mapping and visualization of the landscapes in the domain. As per the best of the knowledge of the authors, there is a dearth of research on the evolution of micro-influencer literature utilizing comprehensive bibliometric analysis using VOSviewer and Biblioshiny, which affirms the originality of the study. The study focuses on addressing this gap with a data-driven approach, identifying thematic clusters, sources, significant contributors, and emerging research areas. A systematic review is “not the end of the road, but the beginning of new journeys”. ^12^ Therefore, the main goal of this study is to explore the development of the knowledge structures of micro-influencer literature with the investigation of key themes while providing future research areas and actionable insights for scholars and practitioners.

This study aims to address the following research questions:

RQ1:
How has the literature on micro-influencers developed?

RQ2:
What are the key themes and research focuses in the existing literature on micro-influencers?

RQ3:
What are the implications for future research on micro-influencers?

The first research question focuses on the development of literature on micro-influencers in the past years, analyzing the empirical papers, authors, and journals. The second research question aims to identify the themes and focuses on the existing literature, and the final research question focuses on identifying implications for future researchers in the micro-influencer domain.

## Methods

The methodology of this study is a systematic literature review (SLR) with a bibliometric analysis focusing on analyzing the research area, “micro-influencers”. This reviews the literature systematically by setting clear exclusion and inclusion criteria. ^13^ Preferred Reporting Items for Systematic Reviews and Meta-Analyses (PRISMA), which is popular among medical research, was utilized as this reporting is lacking in social science studies in this arena. ^14^

Our study employed PRISMA, utilizing a rigorous, systematic, and objective process of selection. Further, the study aims to identify knowledge growth and future research areas in this domain. ^15^ SLRs have gained wide recognition and are suiting for micro-influencer-focused study due to the manageable dataset, and with the wide availability of research articles, proper analysis can be conducted, going beyond simple summarization. ^16^ Transparency and replicability are notable principles of SLRs. ^17^ Publication impact, influence of the authors, keywords, and top journals that published articles are aimed at discovering with the literature review exploration.

### Search strategy

The next stage requires identifying the exact papers for the analysis and launching the scientific query in the database. Hence, the study employed four steps in the PRISMA flow diagram, which are identification, screening, eligibility, and inclusion. At the first step of PRISMA, the Scopus database was selected, and two search strings were utilized: “micro influencers” OR “nano influencers.” Scopus database includes over 20,000 peer-reviewed journal titles. ^18^ This search took place in April 2025. Scopus was picked over Web of Science (WoS) due to the large number of papers covered in Scopus, and 97% of WoS-indexed papers are included in the Scopus domain. ^19^ In the identification stage, 369 articles were identified. The authors identified duplicates and removed them.

The criteria utilized for searching are unbiased and reproducible. At the screening stage, the research adheres to several inclusion and exclusion criteria. Only empirical articles published in journals were selected, excluding book chapters, books, conference papers, reviews, conference reviews, short surveys, research notes, and articles in press. ^20, 21^ With the implementation of this criterion, 160 articles were shown, and further exclusion criteria were set. English articles published between 2020 and 2025, in social sciences, business, management and accounting, economics, econometrics, finance, arts and humanities domains were included. This resulted in downloading 100 papers from the database as a CSV file with title, abstract, author(s), authors’ keyword, number of citations, year, affiliation, sources, and references.

**Figure 1.**
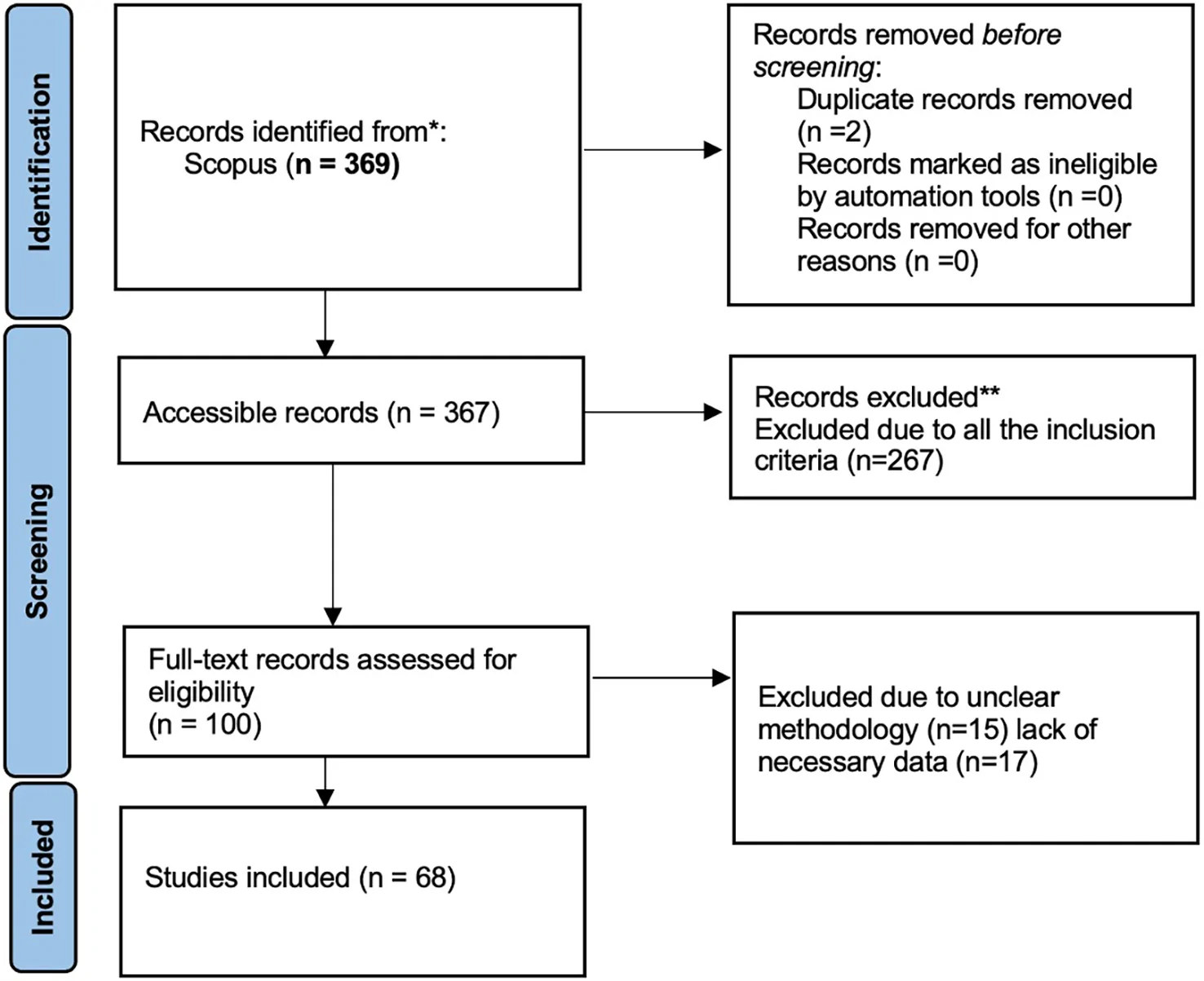
The PRISMA flow diagram for identification of articles from the database. Source: Constructed by the authors.

2020 was set as the base year, considering the post-COVID digital acceleration, increased digital engagement, algorithmic distribution of content, and maturity of the micro-influencer research area. Post-COVID consumer shift toward increased digital engagement, online purchase surge, which influenced the positioning of micro and nano influencers. AI-driven algorithmic platform adoption also started getting popular in emerging social media platforms around this time. 2025 was selected as the ending time to grasp the insights of the recent literature developed. Further, at the screening stage, a total of 32 articles were excluded by the researchers through content analysis, which identified articles with unclear methodology and a lack of necessary details. Each author thoroughly reviewed the articles independently based on the set criteria. Any disagreements occurred with regards to article inclusion were addressed. At the final stage, 68 articles were selected for this study. The process of article selection is depicted in Figure 1. On the selected 68 papers, a descriptive analysis and a cluster analysis were performed. Descriptive analysis focused on identifying publication evolution, specific journals, nationwide contribution, and topics. Citations, along with author collaborations, were also identified. ^22, 23^ In order to enhance the value of the research outcomes, bibliometric analysis is utilized in this critical analysis stage. ^24^ Data was analysed using VOSviewer version 1.6.20 and Bibioshiny (Bibliometrix) via RStudio software desktop version 2023.09.1+494 with the intention of increasing the validity of the results. The VOSviewer analytical tool was utilized to visualize bibliometric networks and clusters through co-occurrence and bibliographic coupling analysis. ^25, 26^ Biblioshiny was utilized to derive the most relevant sources, author production identification, treemap with keywords, trend topic analysis, and finally a three-field plot with authors, keywords, and sources. The latter part of the paper includes clustering followed by a critical content analysis of the papers to form research areas and provide future research directions. The next section presents the main research evidence of this paper.

## Results

The main evidence of the systematic review focused on addressing the aforementioned research questions is presented in this section. The rest of the section covers the selected articles’ bibliometric and content analysis.

### Descriptive analysis

**Figure 2.**
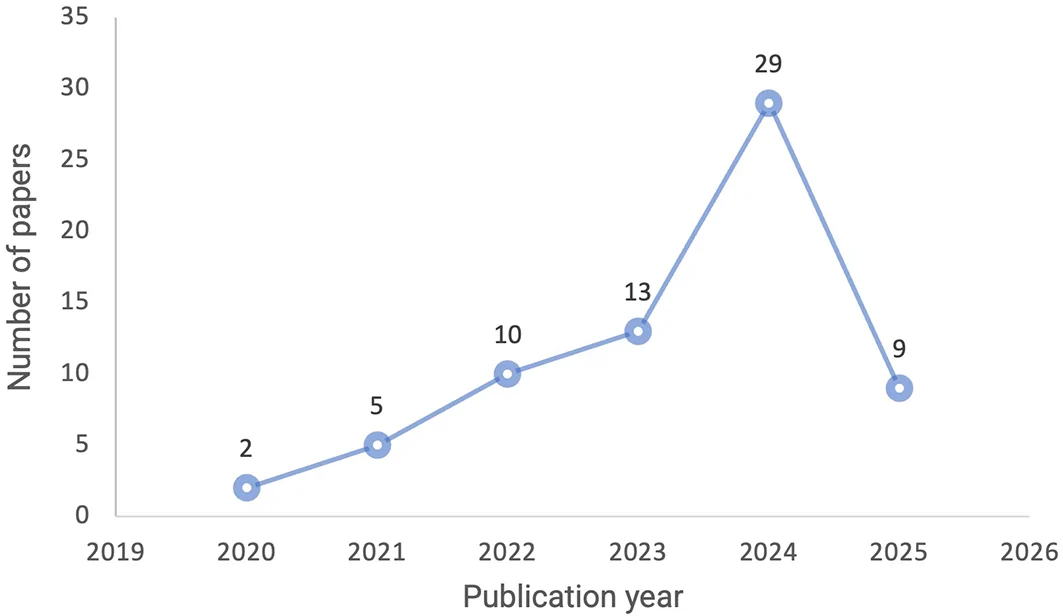
Publication trend between the years 2020 and 2025. Source: Developed by the authors.

*The publications trend* between 2020 and 2025 is depicted in Figure 2. An upward trend can be identified over the years from 2020 up until 2024. In 2020, 2 articles were published, and gradually, in 2024, 29 articles were published. However, the upward trend does not seem to be promising, as up to now only 9 articles have been published. This illustrates the actuality of the research area and the necessity for a thorough investigation into micro-influencer research. This identifies a temporal gap despite the growth. There is a need for continuous and updated research in this area.

**Figure 3.**
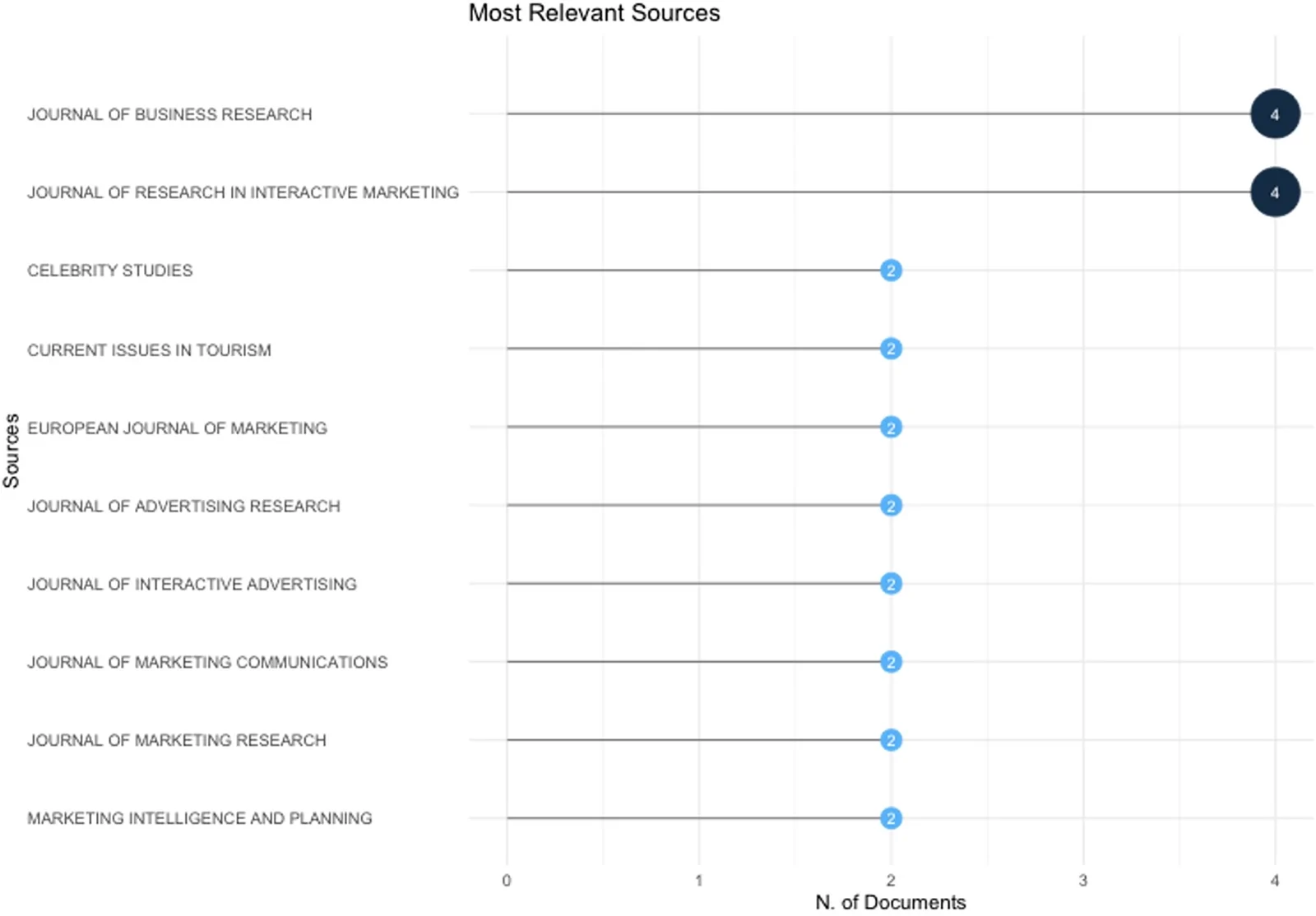
Most relevant sources. Source: Authors generated through Biblioshiny.

In terms of the distribution, which is a crucial metric for researchers, for this study, 68 papers were selected, and this acts as an important sign of the potential for exploring the research subjects. Journal distribution depicts that one to four papers were fragmented across 50 journals. *Journal of Business Research* and *Journal of Research in Interactive Marketing* are the only two journals that have published four papers. As per Figure 3, other journals had about two papers.

This highlights another gap, as the research on this area is highly fragmented and lacks a specialized outlet, which results in slowing theory-building. Future studies should focus on identifying core streams and underexplored areas and should publish in high-impact journals to grow visibility.

*Article categorization* as per the subject areas depicted in Table 1, and it is evident that the leading areas are business, management and accounting, social sciences, computer science and economics, econometrics, and finance. This shows the importance of analysing the impact of micro-influencers in businesses, socially and technologically. Micro-influencer impact on shaping consumer engagement, marketing, and branding could be investigated under the business-related areas. Economic implications of influencer marketing campaigns, computer science involvement with the utilization of data analytics and algorithmic targeting, confirm that the cross-disciplinary focus on studies relating to micro-influencers, which is not only limited to strategic business applications but extends to social behaviours and technological implementation.

**Table 1.** Article categorization based on subject areas.

Subject area	Number of papers
Business, management and accounting	57
Social sciences	46
Economics, econometrics and finance	14
Arts and humanities	4
Computer science	15
Others	6

Source: Developed by authors.

**Figure 4.**
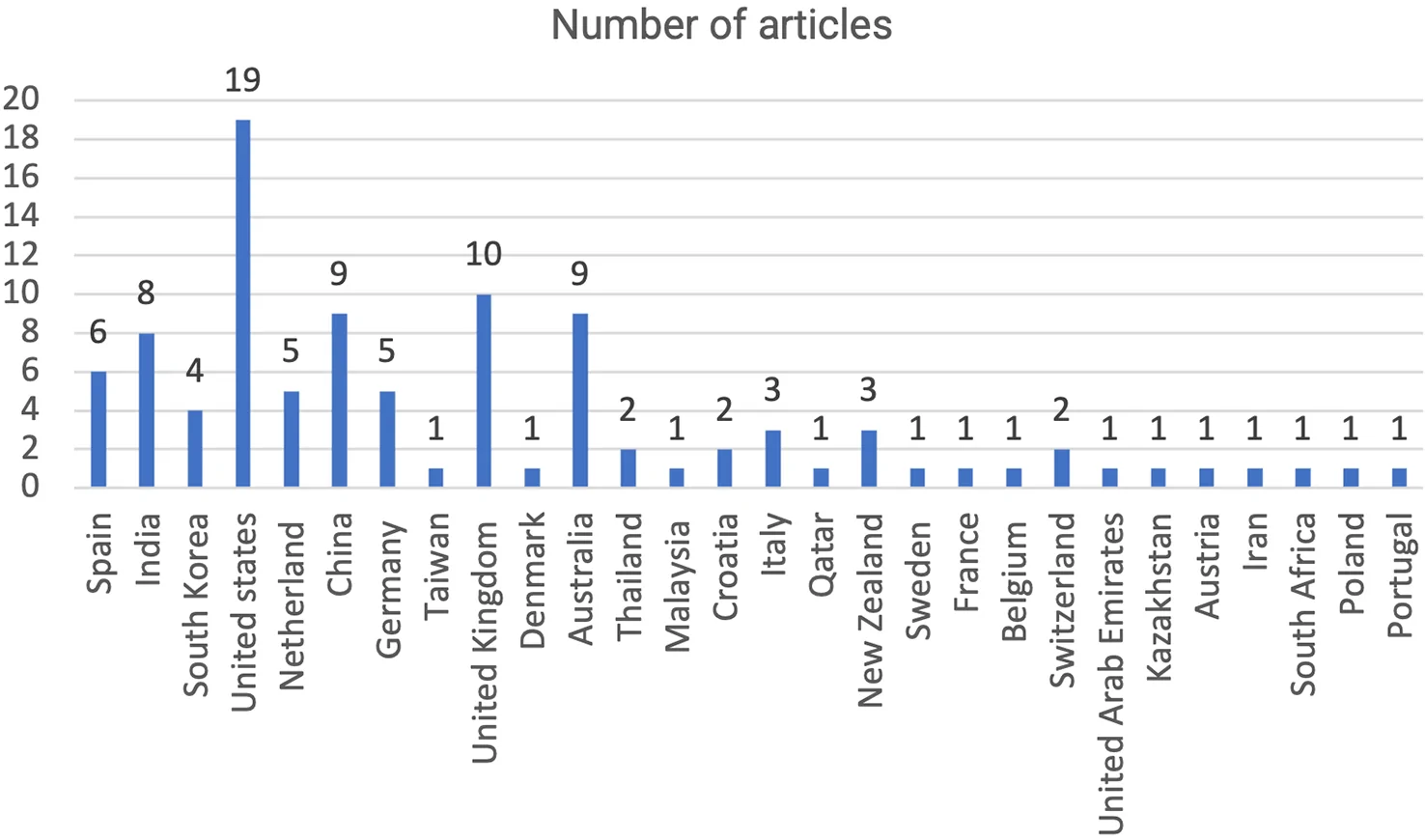
Number of articles per country. Source: Developed by the authors.

**Figure 5.**
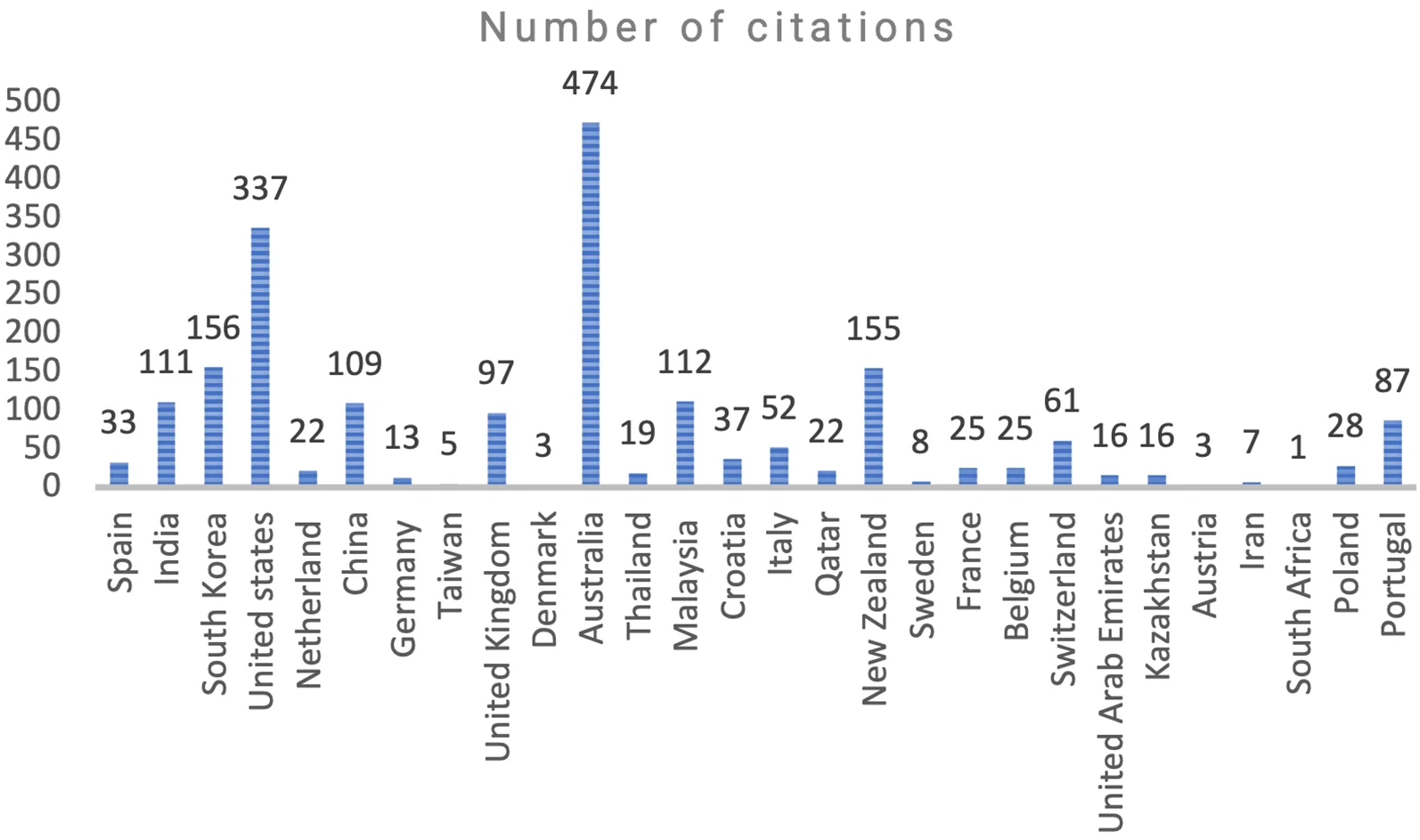
Number of citations per country. Source: Developed by the authors.

**Figure 6.**
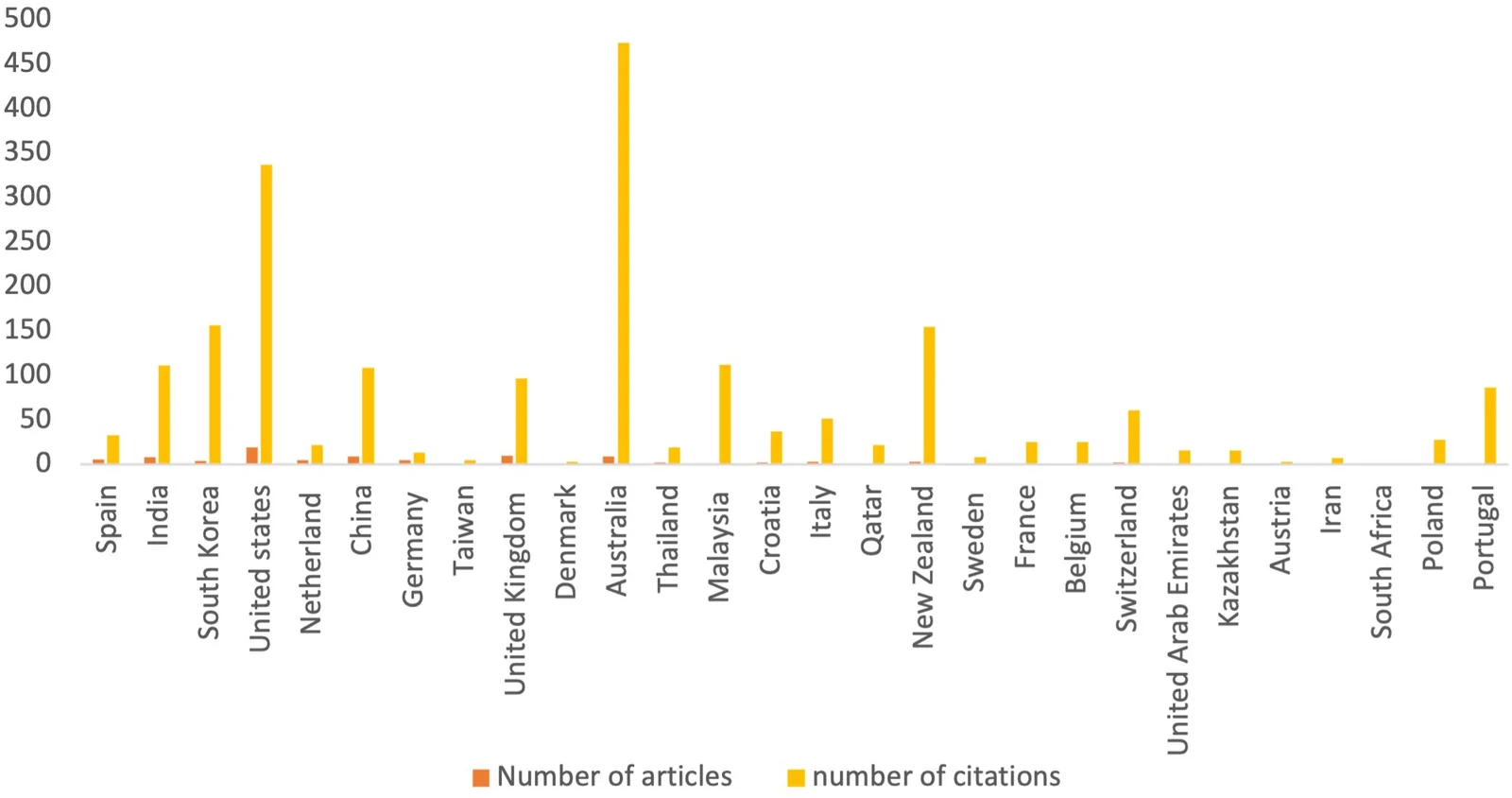
Articles and citations per country. Source: Developed by the authors.

*Geographical analysis of the articles and citations:* to analyse the number of articles published and the number of citations in the monitored time based on the geography, Figures 4, 5, and 6 have been utilized. The authorship, research centre, or base university was considered as the criterion to determine the total number of citations and research articles for each country, and if the research work involved authors from various countries, a point was awarded for each of them. The studies were fragmented across a range of countries, including from United States, the United Kingdom to Malaysia, Taiwan, South Korea, and 28 countries in total have contributed.

The summary of the evidence suggests that the top eight publications belong to the United States, the United Kingdom, Australia, China, India, Spain, the Netherlands, and Germany, as shown in Figure 4. The three countries that topped in terms of publications are, United States with 19 papers, the United Kingdom with 10 papers, China and Australia with 9 papers. India published 8 papers, Spain six, the Netherlands and Germany five each. The citation analysis given in Figure 5 and Figure 6, based on the countries, depicts the top three countries with the highest number of citations: Australia (474), the United States (337), and South Korea (156). Research gaps relating to geographical concentration can be identified as the highest number of citations and publications is skewed towards Western countries and some Asian countries. Many regions, including Africa, the Middle East, and South America (apart from the limited research considered), remain underrepresented. Australia and South Korea, despite having fewer publications than the United States, resulted in high citation counts, showing that high-impact research does not always correlate with volume. Valuable insights could also be derived from fewer article-producing countries, but with highly cited documents.

**Figure 7.**
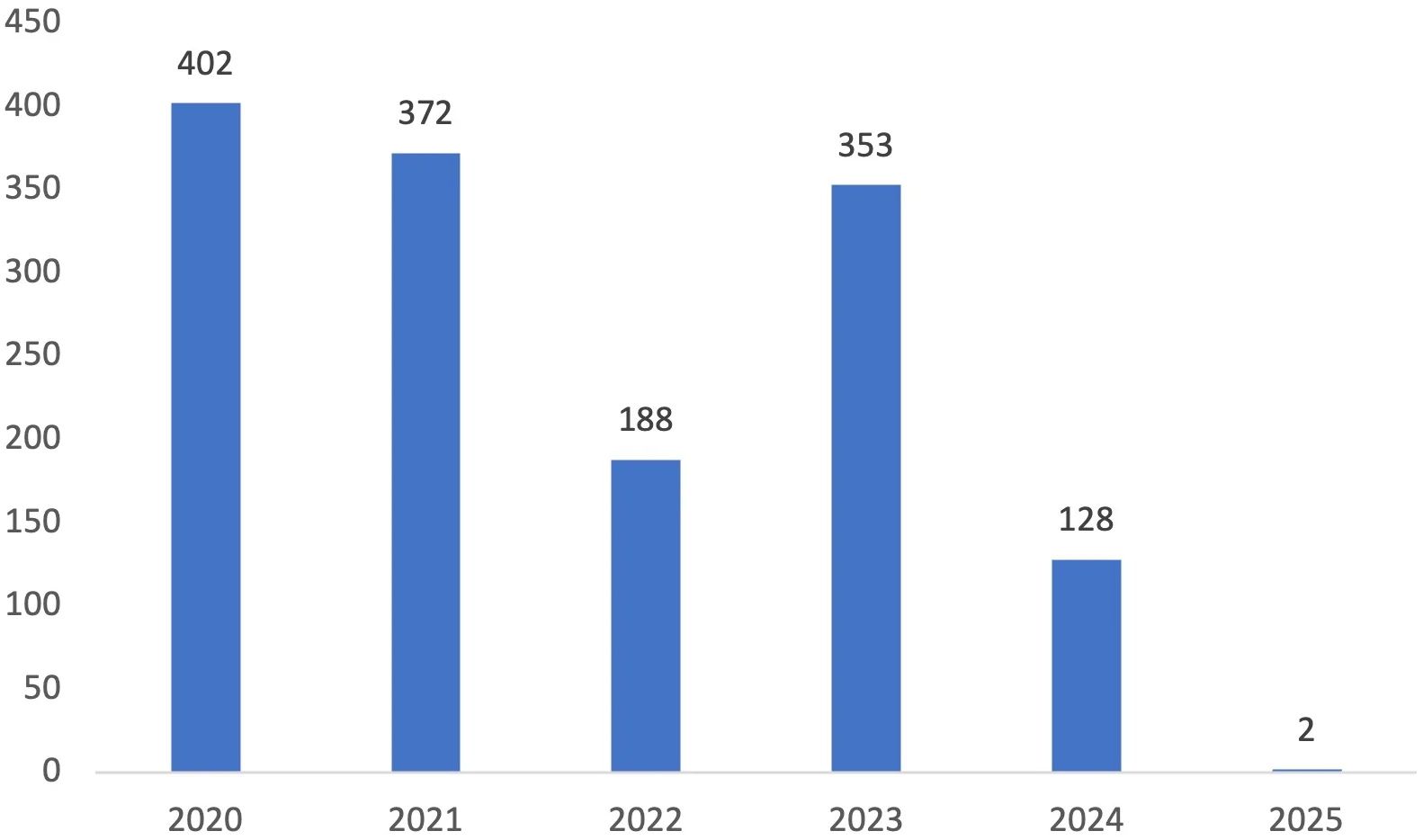
Number of citations received by articles over time. Source: Developed by the authors.

Figure 7 shows the number of citations received by the articles over the considered time. The trend depicts a decreasing nature of the number of citations from the year 2020 to 2022, and again in 2023, this has increased. However, in 2024, this has drastically decreased, and it appears that 2025 will confirm this pattern as well.

Despite the increasing publications over time from 2020 to 2024, citations have not followed the trend. This highlights that publication volume does not correlate with the impact, and future research should aim for theoretically grounded and rigorous studies instead of context-specific exploratory studies.

**Figure 8.**
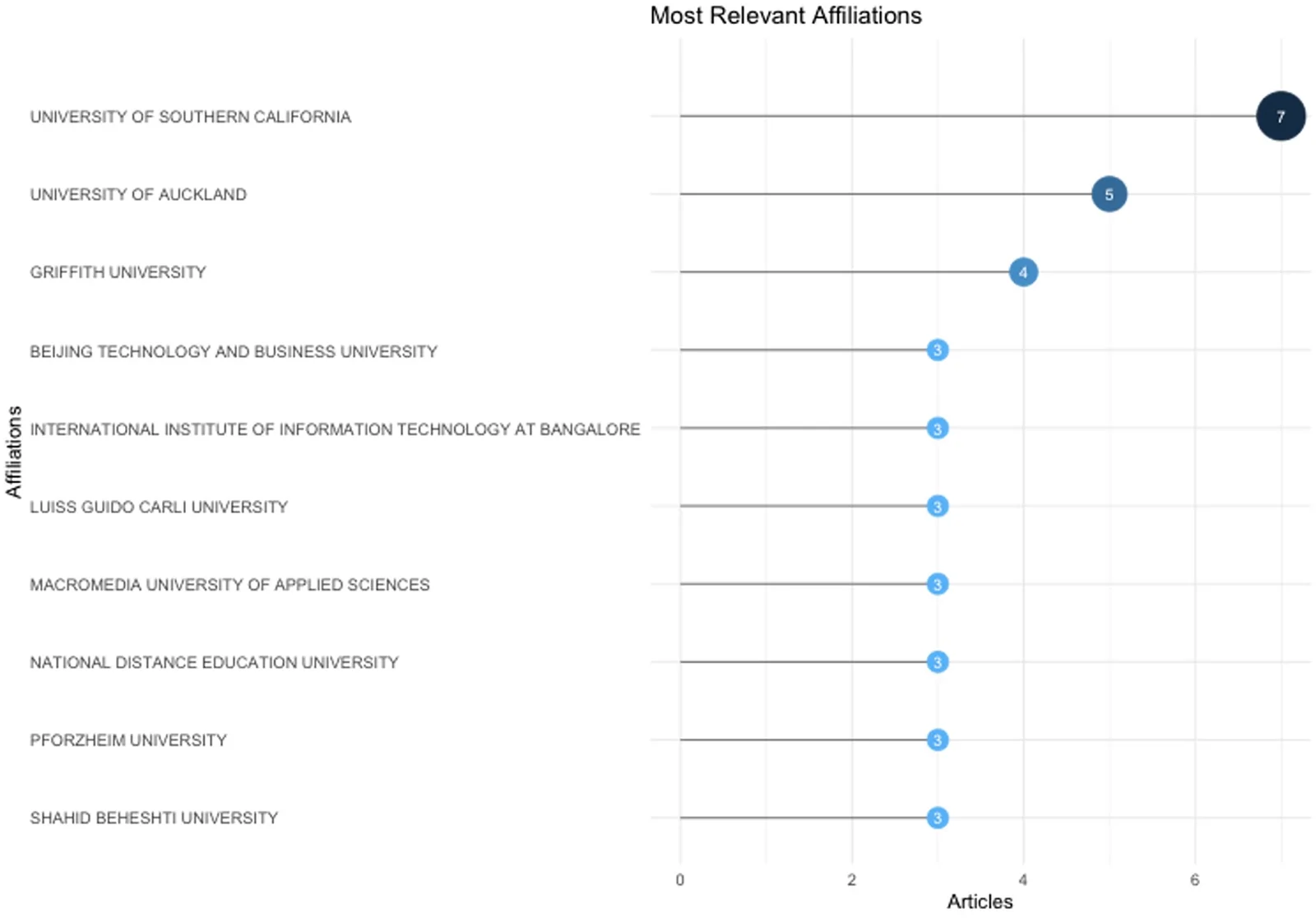
Most relevant affiliations. Source: Developed by the authors through Biblioshiny.

The top three mostly cited papers, which are listed in Table 2, demonstrate citations ranging from 293 to 112. The most cited paper, which gained 293 citations, was published in 2020, whilst the other two were published in 2021, and this demonstrates that the scientific community has greatly valued the efforts. This also suggests that the arena is emerging, and future research should aim to replicate, validate the early findings towards building theoretical robustness. The most relevant affiliations are mentioned in Figure 8, and the University of Southern California, the University of Auckland, and Griffith University can be considered as the top affiliations. This indicates that research is concentrated among a few universities, and this gap could be addressed if future research encourages participation across the globe with a high number of institutions, with the intention to explore cross-cultural studies and diversified methodologies. The dearth of research also showcases the geographical research gaps that limit the generalizability of the findings.

**Table 2.** Top three cited papers.

Authors	Title	Cited by	Source	Country (-ies)
Kay S.; Mulcahy R.; Parkinson J. 2020	When less is more: the impact of macro and micro social media influencers’ disclosure	293	Journal of Marketing Management	Australia
Park J.; Lee J.M.; Xiong V.Y.; Septianto F.; Seo Y. 2021	David and Goliath: When and Why Micro-Influencers Are More Persuasive Than Mega-Influencers	139	Journal of Advertising	South Korea, New Zealand, Australia
Muda M.; Hamzah M.I. 2021	Should I suggest this YouTube clip? The impact of UGC source credibility on eWOM and purchase intention	112	Journal of Research in Interactive Marketing	Malaysia

Source: Developed by authors.

**Figure 9.**
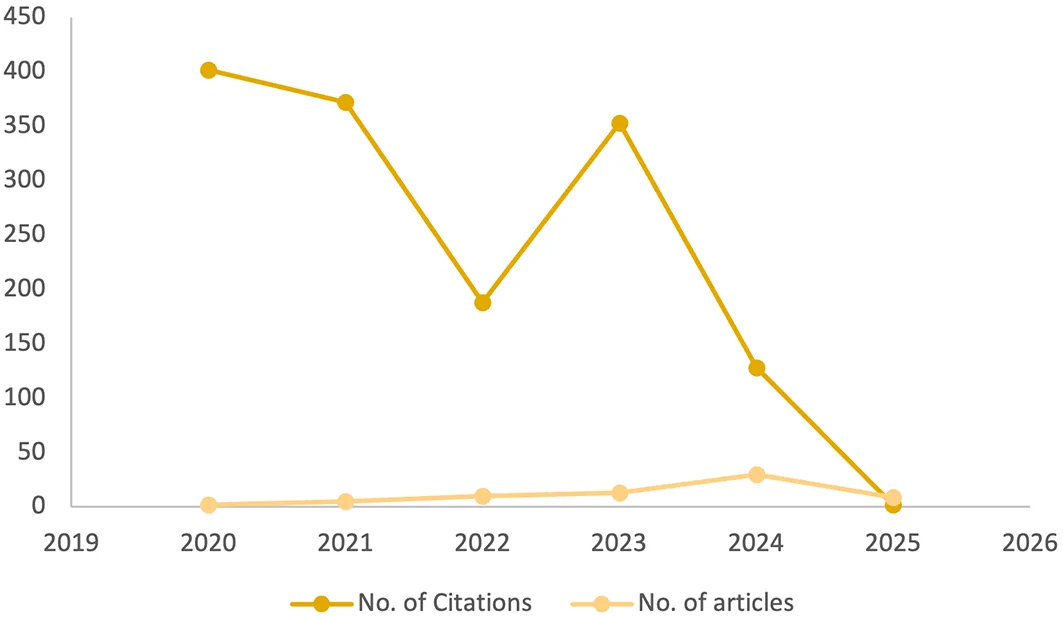
Number of articles and citations gained. Source: Developed by the authors.

**Figure 10.**
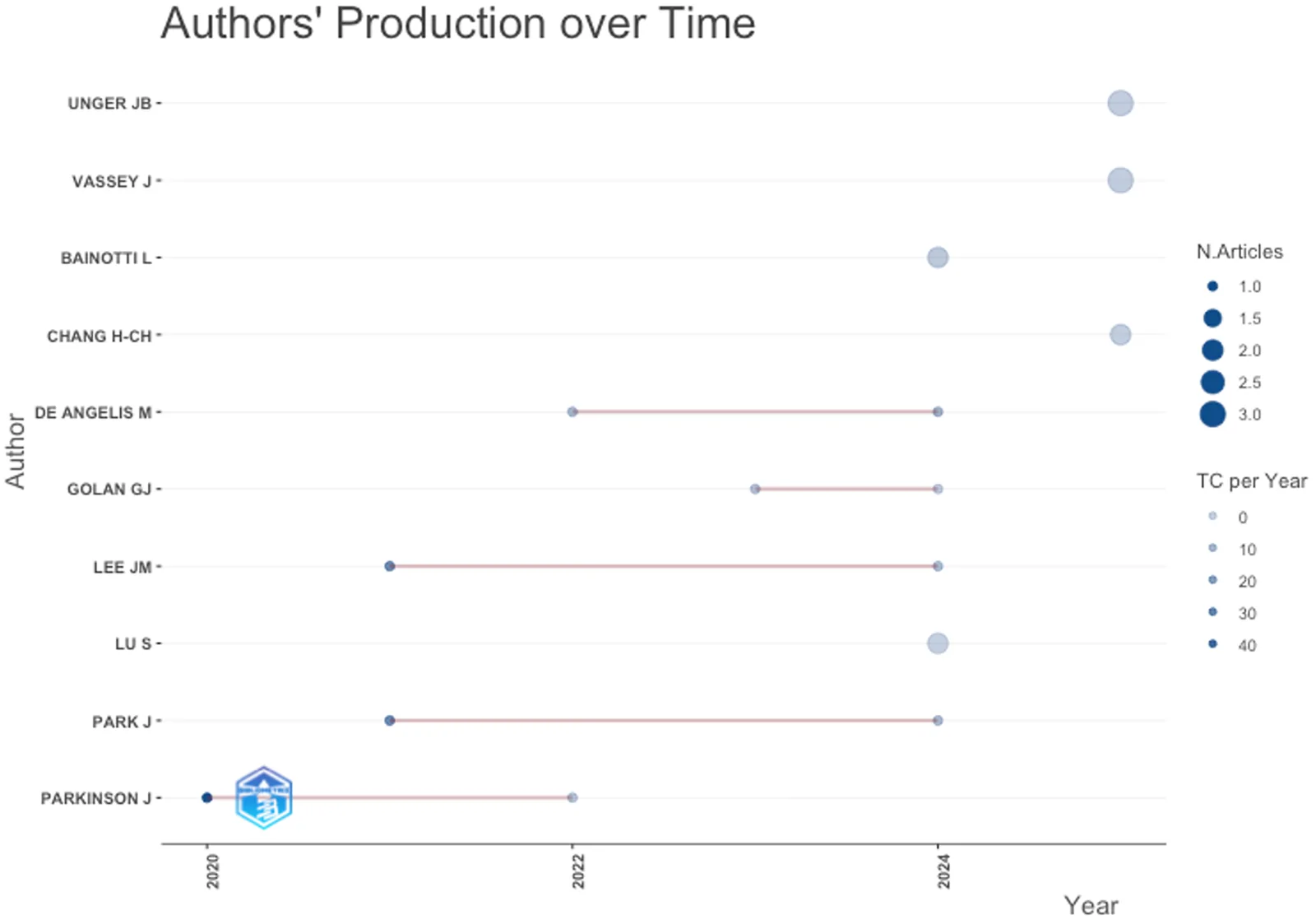
Author's production. Source: Developed by the authors through Biblioshiny.

Figure 9 gives a clear overview of the number of articles published and the number of citations gained from 2020 to 2025. In all the years, the number of citations has exceeded the number of published articles. The peak of the citations was in the year 2020, yet the number of articles in that year remains two. From the years 2020 to 2022, there was a downward trend in terms of citations, and in 2023, citations increased from 188 to 353. However, the citations have shown a downward trend from 2023 to 2025. The number of articles published from 2020 to 2024 has shown an increasing trend, and in 2025, it has decreased. Publication activity of top authors in the domain is depicted in Figure 10. *Unger JB*, *Vassey J*, and *Chang H-CH* were the most productive authors who published many papers in 2024 that contributed towards a notable citation impact. Consistent involvement has been shown by *De Angelis M*, *Golan GJ*, and *Lee JM* by publishing from 2021 to 2024. *Lee JM* has been an active contributor across four years. *Parkinson J*, *Park J*, and *Lu S* have been able to make contributions over short periods, while *Parkinson J*, who started in 2020, continued through 2022. Even though authors have few publications, certain authors have achieved moderate citation influence. A diverse and focused group of researchers has shaped this stream.

A very small number of authors appeared in 2020 and 2021, indicating a gap in baseline studies in the arena. To build a more complete understanding of micro-influencers, future research should be carried out to uncover understudied variables, especially in qualitative and exploratory terms. Short-lived research involvement can be identified with the limited multi-year productivity with the publication concentration in 2024. Researchers should design multi-phase and longitudinal studies targeting multi-year productions. Future studies should consider long-term collaborations.

**Figure 11.**
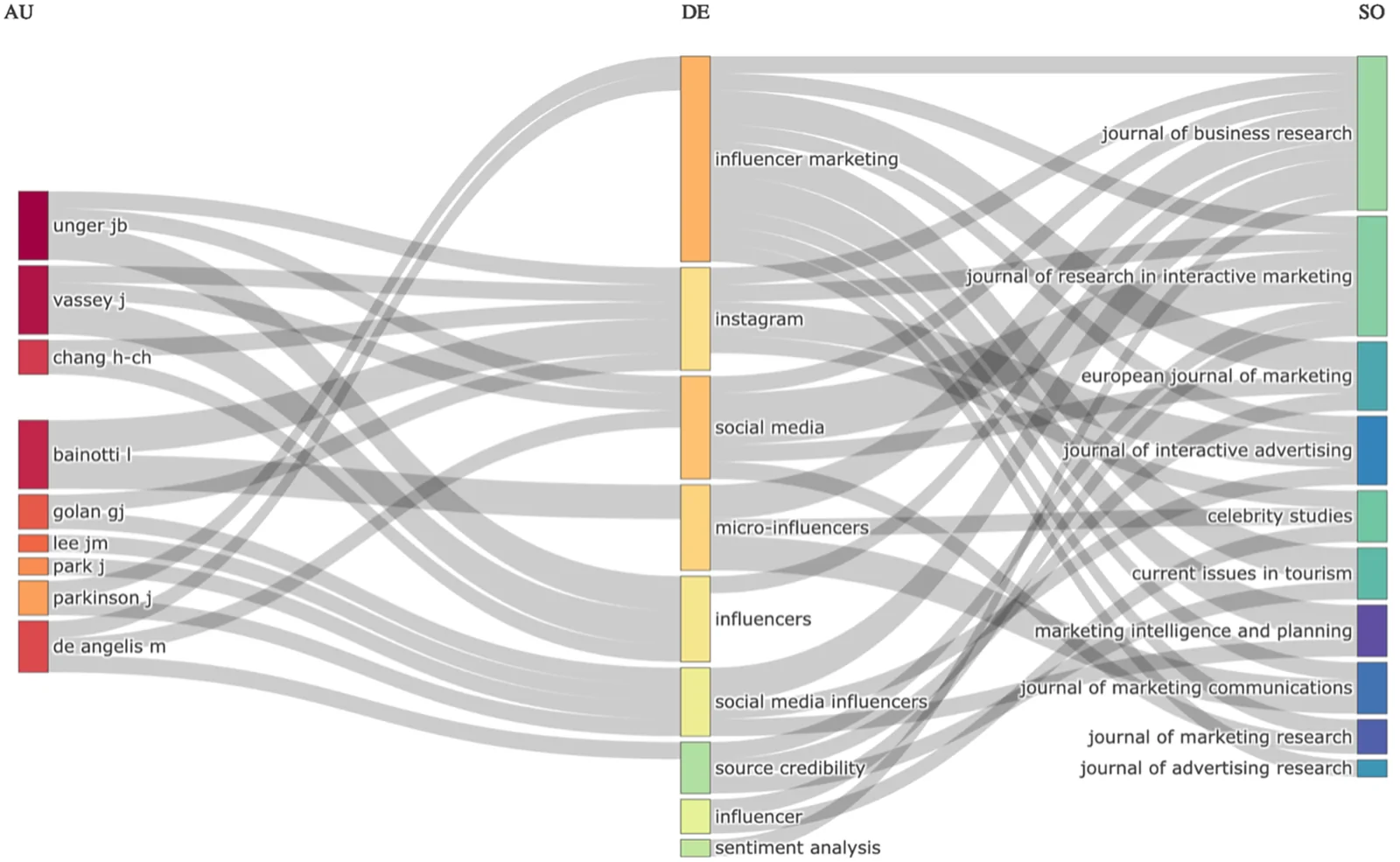
Sankey Diagram. Source: Developed by the authors through Biblioshiny.

In Figure 11, the Sankey diagram is used to visualize the relationship between the most relevant authors, keywords, and sources. Each bar shows the frequency and relevance, and the size shows the contribution. One of the central keywords being micro-influencers, depicts the strong focus of research in this area. Certain authors, including Golan GJ, Vassey J, and Chang H-CH, have published articles relating to micro-influencers while being connected to themes such as ‘influencer marketing’, ‘Instagram’, and ‘social media’. These have been published in the Journal of Business Research, Journal of Research in Interactive Marketing, and the European Journal of Marketing. Research with overlapping keywords suggests collaborative trends. Even among closely related keywords such as ‘social media influencers’, ‘influencers’, and ‘influencer marketing’, the specific emergence of ‘micro-influencers’ confirms the trending and growing research interest in this niche area. This provides a clear understanding of the prominent authors, sources, and keywords.

*Topics and common keywords*

VOSviewer and Biblioshiny were used for the keyword occurrences to retrieve the most common topics and keywords initially. In order to conduct the keyword analysis, the keywords utilized by the authors and the publishers were considered. A large number of papers can be analysed while focusing on the depth of the small concepts. ^27^

**Figure 12.**
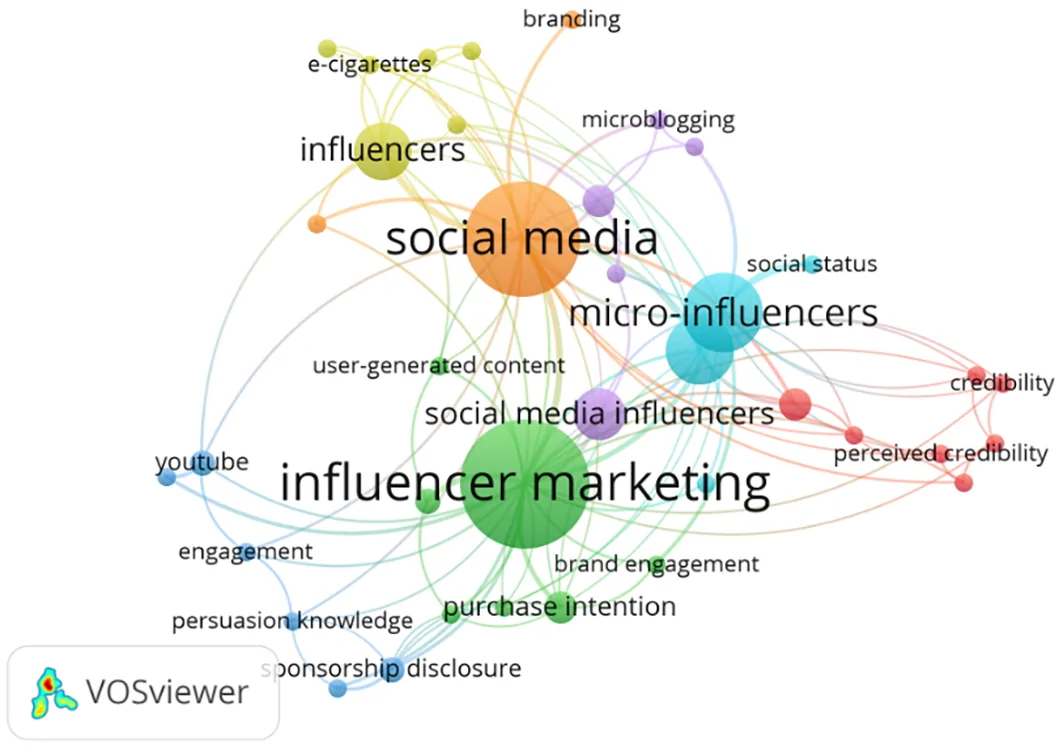
Authors' keywords' clusters. Source: Developed by the authors through VOSviewer.

The Keywords were retrieved from the chosen articles, which were later subdivided. ^28^ The keyword subdivision, as per the co-occurrence using VOSviewer, resulted in seven clusters. In the process of extracting the most common keywords, the threshold was set to at least two times, and the unit of analysis was selected as the author’s keywords. The formed clusters, along with the keywords, are depicted in Table 3 and Figure 12. The keyword “influencer marketing” was identified as the most recurrent keyword, with the occurrence of 22 times, then “social media” and “micro-influencer” were identified. These top-three keywords are in different clusters, in particular clusters 2, 6, and 7. Micro-influencers are a part of the wider social media marketing domain, with studies focusing on comparisons with different types of influencers. Hence, the identification of influencer marketing as the most occurring keyword is consistent with the studies, such as Walter et al. ^5^ A clear gap can be identified as certain constructs that have a clear link toward social media micro-influencers, like self-branding, parasocial intimacy, and algorithmic visibility, were not visible in this analysis. This suggests that despite the link, these areas remain underexplored, and future studies should focus on this arena.

**Table 3.** Author’s keywords occurrence.

	Keywords	Occurrences
Cluster 1 (7 items- Red)	Credibility	2
	Influencer	4
	Perceived credibility	2
	Persuasion	2
	Similarity	2
	Social media influencer	2
	Trust	2
Cluster 2 (7 items- Green)	Brand engagement	2
	Influencer marketing	22
	Micro-influencer	2
	Parasocial relationship	2
	Purchase intention	4
	Source Credibility	3
	User-generated content	3
Cluster 3 (6 items-Blue)	Engagement	2
	Influencer type	2
	Persuasion knowledge	2
	Social networks	2
	Sponsorship disclosure	3
	YouTube	3
Cluster 4 (6 items-Light green)	E-cigarettes	2
	Influencers	8
	Misinformation	2
	Nicotine	2
	Social network	2
	Tiktok	2
Cluster 5 (5 items-Violet)	Microblogging	2
	Nano-influencers	2
	Natural language processing	2
	Sentiment analysis	4
	Social media influencers	7
Cluster 6 (4 items- Sky Blue)	Covid-19	2
	Instagram	10
	Micro-influencers	12
	Social status	2
Cluster 7 (3 items-Orange)	Branding	2
	Micro influencer	2
	Social media	19

Source: Developed by authors.

**Figure 13.**
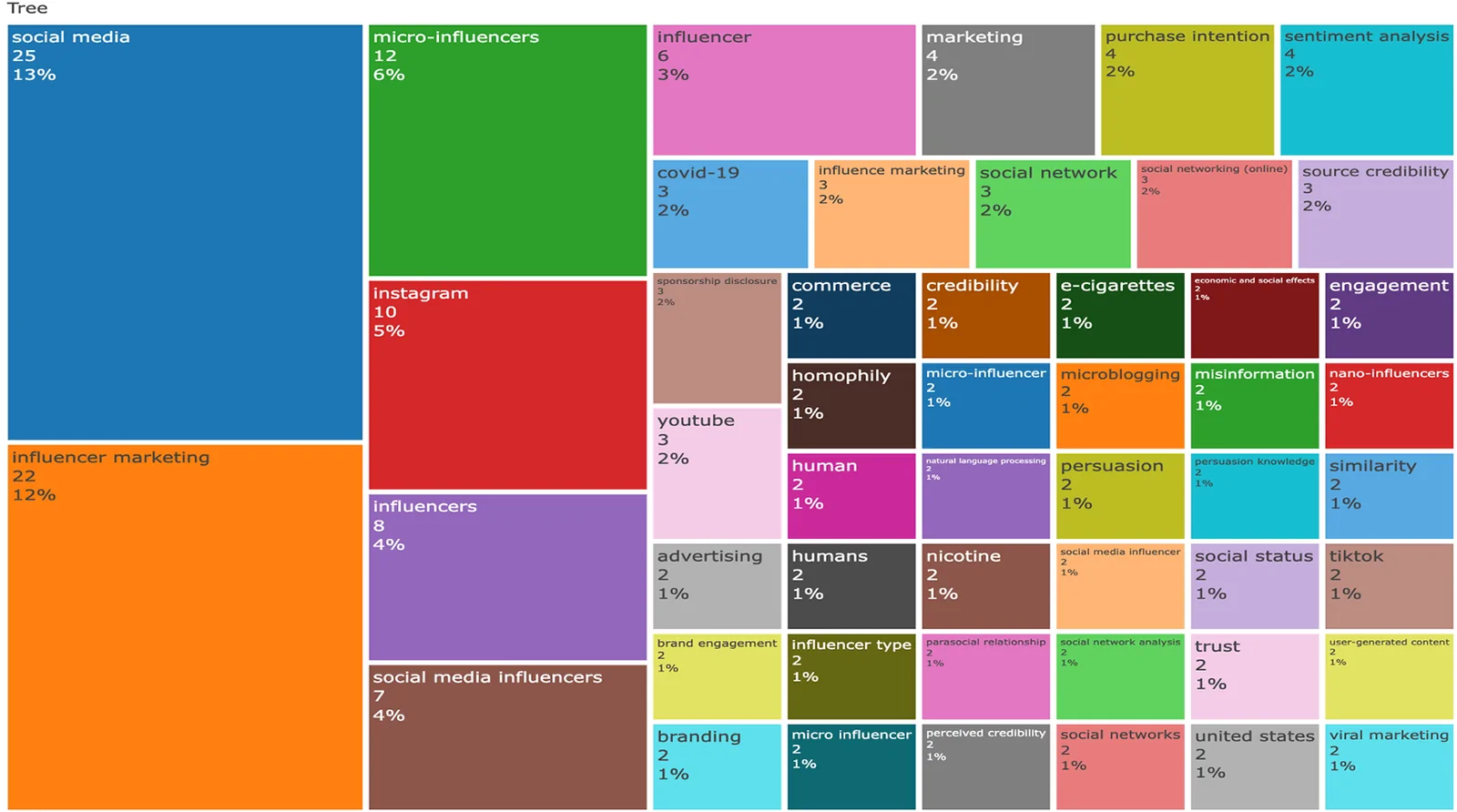
Treemap. Source: Authors generated through Biblioshiny.

Further, with *Biblioshiny*, a Treemap was used in Figure 13 to provide a clear representation of the keywords, as treemaps effectively represent the keyword repetition and hierarchical structure with stacked rectangles. The value decides the area of the rectangle. ‘Micro influencers’ was the third most frequently occurring keyword with 12 mentions. Key domains in the research area were depicted from this, and a social media platform, which is Instagram, was the 4th frequently occurring keyword in the study. This is consistent with Liao et al., ^29^ as these studies specifically focused on Instagram.

**Figure 14.**
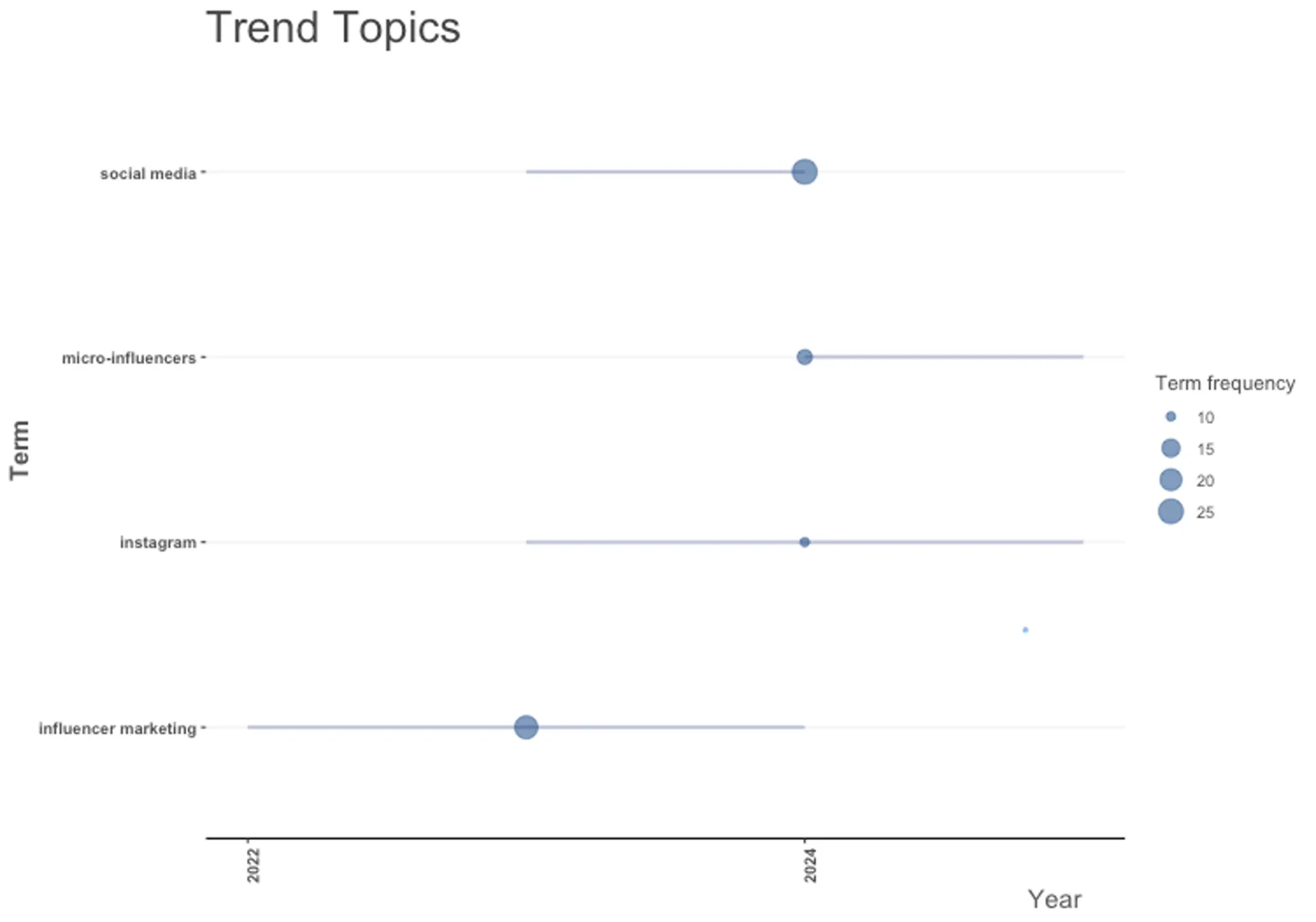
Trend Topics Analysis. Source: Authors generated through Biblioshiny.

Trend topic analysis was conducted using *Biblioshiny* with the intention of identifying the most frequently discussed topics in the domain, as shown in Figure 14. Minimum word frequency was set at ten. ‘Influencer marketing’ was a prominent topic starting from 2022, along with ‘social media’, ‘Instagram’, and ‘micro-influencers’ in the years 2023 and 2024. This indicates the niche and targeted marketing strategies focusing on ‘micro-influencers’ and ‘Instagram’. The recent trend topics indicate the dominance of the Instagram studies, and a clear gap can be identified with the underrepresentation of emerging platforms such as TikTok and Twitch. Future studies should focus on these platforms. Further, future research should focus on theory building towards platform-specific visibility.

*Clustering and content analysis*

**Figure 15.**
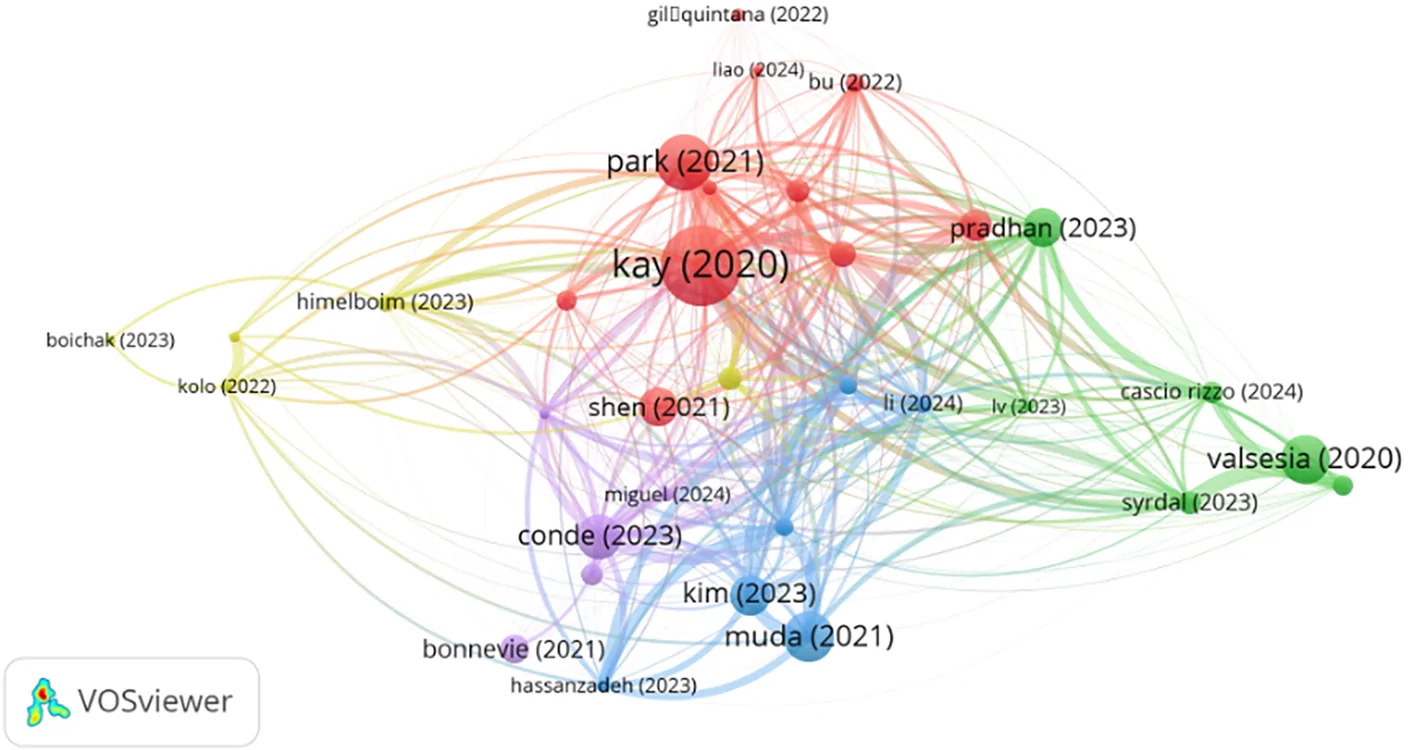
Clusters formed with intersected literature. Source: Developed by the authors through VOSviewer.

In order to evaluate the relatedness, the articles that share similar references have been considered under the clustering in bibliographic coupling. ^30, 31^ Bibliographic coupling was conducted with the use of VOSviewer. The unit of analysis was selected as documents, the threshold was a minimum of five citations, and the fractional counting method was followed. This analysis resulted in 33 connected papers because only papers with at least five citations were selected and generated five clusters. The five clusters generated from bibliographic coupling are depicted in Figure 15, and it is vital to comprehend that the strength of the closeness is related to the quantity of shared bibliographies that occur in the article.

*Content analysis*

Rigorous content analysis has been performed with the objective of thoroughly grasping the primary developing research areas. After carefully reading all the publications, the researchers conducted the content analysis in order to categorize them into research areas. The analysis categorized articles into five research areas, and each of these synthesized the corpus of knowledge. The identified five research areas are as follows: Sponsorship disclosure, influencer types and decision making: a multilevel analysis, Strategic language and micro-influencer engagement, Behavioural outcomes and interpersonal dynamics of micro-influencer marketing, micro-influencers in identity construction and professionalization, and micro-influencers as persuasive actors in niche domains. The articles belonging to the aforementioned research areas are given in Table 4, which is included in the repository. ^32^ Research area 1 comprises the highest number of literature among all the clusters, spanning from 2020. Themes of the articles covered in the cluster are converging predominantly on sponsorship disclosure, influencer marketing, influencer type, and responses of the consumers, while emphasizing micro-influencers. One area identified in the cluster was comprising studies focusing on the perceived consumer behaviour pertaining to sponsorship disclosure and influencer credibility. Explicit sponsorship disclosure resulted in increasing the behaviour of value co-creation, ^33^ while the investigations related to the transparency of sponsorship linking to the decision-making stages depicted a significant impact of trust and sponsorship transparency, highlighting the problem recognition to post-purchase in the decision-making process. ^34^ Transparency and communication have resulted in boosting consumer credibility among micro-influencers, along with having a positive impact on purchase intention. The impact of disclosure on brand evaluations and the trust among consumers has also been investigated. ^35, 36^

**Table 4.** Research papers based on the research areas.

Research areas	Year	Citations	Title
Sponsorship disclosure, influencer types and decision making: a multilevel analysis (11 papers)	2022	15	Influencer marketing: sponsorship disclosure and value co-creation behaviour
	2023	9	Which decision-making stages matter more? Influencer’s perceived credibility, sponsorship and moderating role of trust
	2022	8	Nano-Influencers Edutubers: Perspective of Centennial Generation Families in Spain
	2022	45	Sponsorship Disclosure of Influencers – A Curse or a Blessing?
	2020	293	When less is more: the impact of macro and micro social media influencers’ disclosure
	2024	5	Does the verified badge of social media matter? The perspective of trust transfer theory
	2021	139	David and Goliath: When and Why Micro-Influencers Are More Persuasive Than Mega-Influencers
	2021	18	How to convert Millennial consumers to brand evangelists through social media micro-influencers
	2022	31	Consumers’ self-reported and brain responses to advertising post on Instagram: the effect of number of followers and argument quality
	2021	66	A persuasive eWOM model for increasing consumer engagement on social media: evidence from Irish fashion micro-influencers
	2022	25	Managing the Transparency Paradox Of Social-Media Influencer Disclosures: How to Improve Authenticity and Engagement When Disclosing Influencer-Sponsor Relationships
Strategic language and micro-influencer engagement (6 papers)	2024	21	How High-Arousal Language Shapes Micro- Versus Macro-Influencers’ Impact
	2023	5	Macro-influencers or meso-influencers, how do companies choose?
	2023	69	Influencer marketing: When and why gen Z consumers avoid influencers and endorsed brands
	2023	25	Influencer marketing and the growth of affiliates: The effects of language features on engagement behavior
	2024	19	Mega or Micro? Influencer Selection Using Follower Elasticity
	2020	109	The Positive Effect of Not Following Others on Social Media
Behavioural outcomes and interpersonal dynamics of micro-influencer marketing (6 papers)	2023	7	Who one is, whom one knows? Evaluating the importance of personal and social characteristics of influential people in social networks
	2024	15	Influencer marketing in the promotion of tourist destinations: mega, macro and micro-influencers
	2023	74	Social media influencers as human brands: an interactive marketing perspective
	2024	14	How micro- (vs. mega-) influencers generate word of mouth in the digital economy age: The moderating role of mindset
	2021	112	Should I suggest this YouTube clip? The impact of UGC source credibility on eWOM and purchase intention
	2024	13	Parasocial relationships with micro-influencers: do sponsorship disclosure and electronic word-of-mouth disrupt?
Micro-influencers in identity construction and professionalization (5 papers)	2024	5	How conspicuousness becomes productive on social media
	2023	5	Mapping the Russian Political Influence Ecosystem: The Night Wolves Biker Gang
	2023	14	A Social Network Approach to Social Media Influencers on Instagram: The Strength of Being a Nano-Influencer in Cause Communities
	2022	5	Formal Professionalization of early-stage Social Media “Influencers”—attitudinal Drivers and Their Relation to Personality Traits
	2023	24	Instagram Influencers in Health Communication: Examining the Roles of Influencer Tier and Message Construal in COVID-19-Prevention Public Service Announcements
Micro-influencers as persuasive actors in niche domains (5 papers)	2021	37	Social media influencers can be used to deliver positive information about the flu vaccine: Findings from a multi-year study
	2023	87	Micro, macro and mega-influencers on instagram: The power of persuasion via the parasocial relationship
	2024	5	Can residents engage potential tourists as ‘micro’ and ‘nano’ influencers?
	2024	8	Self-branding and content creation strategies on Instagram: A case study of foodie influencers
	2022	22	Exploring the synergy between nano-influencers and sports community: behavior mapping through machine learning

Source: Developed by authors.

The second group identified among the cluster focuses on the persuasive impact and the characteristics of micro-influencers. In the investigation among millennials, it is evident that evangelism and brand engagement were boosted as a result of the emotional connection built by micro-influencers, along with the authenticity. ^37^ Micro-influencers were evident to be more persuasive in terms of authenticity, when compared with mega-influencers, when it comes to the hedonic context of products. ^38^ Trust transfer theory has been a crucial theory in the identification of the effectiveness of verified badges among micro-influencers to enhance trust and behavioural intentions. ^29^ The third group identified among the clusters focuses on the consumer engagement and content factors, to be precise, which explores the content-specific engagement with the micro-influencer eWOM ^39^ and explorations in various contexts involving educational as well with regards to nano-influencers and their follower interaction. ^40^ Authenticity can be regained strategically via carefully managed disclosures ^41^ while neuroscientific experiments are also involved in the validating process of argument quality and source credibility in influencer posts’ responses. ^42^ Overall, this cluster depicts an understanding of multifaceted concepts built around micro-influencers’ roles in building consumer trust, perceptions, and concepts such as disclosure of sponsorships.

Findings in this cluster relate to sponsorship transparency as a factor that shapes credibility. However, the contradictory results depict that weakening or strengthening the authenticity perceptions is context-specific. This inconsistency gap identifies an unsettled transparency-authenticity paradox. Future research should focus on specifically identifying which boundary conditions, like product involvement or the strength of influencer-follower relationships, result in fostering trust or eroding it. Research area 2: This research area is equipped with literature that focuses on behavioural and strategic dimensions of influencer marketing with regard to micro-influencers. The articles included in this area focus on linguistic techniques, generational-based engagement patterns, and strategies for selecting suitable influencers. With regards to the linguistic features, the language used by the type of influencers results differently; when high arousal language is used by micro-influencers, increased engagement was depicted, whereas if the same was used by macro-influencers, trust and interactions resulted in diminishing unless mitigated by informational framing. ^43^ The strength of the persuasiveness associated with affiliated marketing content is influenced by linguistic cues that are said to have an impact on cognitive processing routes. ^44^ Another focus of this research area is decision making and influencer selection, and this is mainly aimed at exploring the follower size, reach, and cost. In the course of making a decision between picking micro and mega influencers, the follower elasticity of impressions (FEI) framework was introduced. ^45^ This framework acts as a determinant when making decisions focused on visibility and efficiency, and if trade-offs are necessary. Accumulating micro-influencers in low-familiarity brands has been suggested by the evolutionary game theory simulations. ^46^ The final focus of this research area is on generational-based engagement patterns and consumer responses, especially focusing on Generation Z. Brand avoidance behaviour is evident among Gen Z consumers, resulting from either perceived manipulation or lack of authenticity. The “influencer avoidance” behaviour highlights the moral aspects of digital marketing, and it is evident that macro influencers that are heavily controlled by brands are perceived in a negative manner by Gen Z consumers. ^47^ In order to build a strong trust among these Gen Z consumers, the influencers should have subtle platform behaviours, including influencers following fewer people, which depicts trust and autonomy, which ultimately results in strong trust and engagement. ^48^

Considering the studies in this area, a clear gap in the under-theorization of narrative and rhetorical aspects of influencer language is identified. Future studies should focus on how storytelling and the tone affect brand connection and perceived sincerity, linking to emotional contagion and identity performance frameworks.

Research area 3: This research area focuses on the effectiveness of micro-influencer marketing in terms of interpersonal traits, emotional bonding, and trust. Followers’ perception of influencers’ personal and social attributes constructs marketing and opinion leadership outcomes is emphasized in this area. Para-social interaction was identified as a mediating factor for opinion leadership, while personal characteristics, including openness, interpersonal competence, and exhibitionism, were considered key factors driving opinion leadership. ^49^ Homophily, attractiveness, and social presence result in building emotional bonds based on the human brand perspectives and attachment theory. The emotional bonds built among the influencers and followers result in loyalty and advertising credibility. ^50^ Parasocial relationships are crucial with regard to micro-influencers and negative eWOM and sponsorship disclosure disrupt and result in the reduction of engagement and purchase intentions. ^51^ In terms of word-of-mouth generation, ‘mindset’ is considered a moderator; endorsements of micro-influencers were perceived as more trustworthy by a set of consumers who are said to have a growth mindset. ^52^ Purchase intention and eWOM are significantly influenced by perceived source credibility of user-generated content (UGC) in platforms such as YouTube. Further, these are also mediated by homophily over traditional voices of the brands. ^53^ In tourism, which is a niche domain, customer attitudes toward travel destinations have been influenced by micro-influencer post engagement and credibility. ^54^

As the studies are predominantly cross-sectional, the temporal evolution of relationships or trust in this arena remains underexplored. Experimental and longitudinal study designs should be utilized to identify the fluctuations of engagement over time. Findings could be explained with brand community and relationship marketing models.

Research area 4: This research area focuses on micro-influencers and nano-influencers beyond traditional product endorsement while emphasizing social status, cause-related engagement, public communication, and professional identity. Veblen’s conspicuous consumption theory is utilized to explain self-branding followed by micro-influencers as productive strategies in order to construct signalling social status. ^55^ In the early stages of growth, professionalization plays a major role in brand collaboration, and this, combined with brand orientation, entrepreneurial orientation, and personality traits, results in a formal market entry. ^56^ Exploration in the veganism communities relating to nano-influencers emphasized structural power in chambers and capabilities for bridging, spreading the cause-based influence and messages. ^57^ Homophily plays a major role in enhancing behavioural intentions, and this was shown during COVID-19, relating to health communication. ^58^ Utilizing micro-influencers in a socio-political context to gain benefits out of various political systems can be identified orientation. ^59^

Though the studies focus on self-branding and entrepreneurial positioning, ethical implications and labour economics remain underexplored. Future studies could explore this arena with the integration of digital labour theory and identity work perspectives.

Research area 5: This research area focuses on micro and nano influencers in community-centric domains, and the power of influencer communications is discussed. Utilizing micro-influencers in larger campaigns, such as flu vaccinations for African American and Hispanic populations in the US, depicted favourable results. This depicts that micro-influencer-generated content has resulted in making changes to social norms, especially in sensitive areas such as health. ^60^ Perceived popularity and credibility of micro-influencers result in a high chance that their followers will adopt the given recommendation under the mediating role of parasocial relationship. ^61^ In the tourism industry, scholars have identified that when micro-influencers contribute as local ambassadors in marketing destinations, they tend to build place identification and emotional solidarity among potential visitors. ^62^ Among food influencers, who produce Instagrammable content marketing efforts, are explored via qualitative methods. ^63^ Machine learning techniques have been utilized in analysing the efforts of nano-influencers in modulating sports communities and cybersecurity. ^64^

As the studies show micro-influencers in different industrial contexts, this showcases an increase in societal relevance. A clear gap is shown in theoretically synthesising micro-influencer credibility to behaviour change. Prosocial influence models could be developed focusing on mobilizing social good while reconsidering communication boundaries.

## Discussion

### Implication 1: How has the literature on micro-influencers developed?

Literature focusing on micro-influencers is relatively young, as per the SLR exploration. Among the years looked into in the analysis, initially in 2020, only two papers were published between 2022 to 2024, an upward trend of publications, promising the research area is gaining popularity across marketing, tourism, health, and communication domains. Earlier studies focused on examining various concepts related to micro-influencers descriptively; however, later studies focused on leading investigations towards authenticity, trust, platform-specific practices, and consumer engagement. Considering the authorship and geographic analyses, the arena is fragmented with underrepresentation of certain regions and limited collaborations across the globe. Though the research domain is growing, a gap can be identified related to theoretical consolidation. Future research should focus on theory development by cumulating psychological, communicational, cultural, and digital perspectives by involving isolated findings.

### Implication 2: What are the key themes and research focuses in the existing literature on micro-influencers?

The key thematic clusters identified as per the bibliometric coupling involves exploration of five research areas: 1) Sponsorship disclosure, influencer types and decision making: a multilevel analysis. 2) Strategic language and micro-influencer engagement. 3) Behavioural outcomes and interpersonal dynamics of micro-influencer marketing. 4) Micro-influencers in identity construction and professionalization. 5) Micro-influencers as persuasive actors in niche domains. Bibliometric mapping and content analysis of the full papers have resulted in a thorough analysis identifying contributions of each cluster and gaps. The main focus among the micro influencer literature is on the capabilities of branding and marketing with regard to authenticity, follower trust, opinion leadership, decision-making, perceived credibility, engagement frameworks, and sponsorship disclosure. ^50, 51, 61^

However, the analysis identified several gaps that need to be addressed. Contradictory findings were identified concerning the sponsorship disclosure; this needs to be validated clearly in future studies. The overemphasis on one specific social media platform, “Instagram,” was visible, labour economics and identity dimensions were underexplored, and a dearth of theoretical synthesis among the wide advertising and communication theory and the behaviour of micro-influencers. The analysis suggests uneven yet rich knowledge development. Researchers should focus on answering under what basis micro-influencers can influence outcomes and why, going beyond detailing effects. This can be achieved with comparative designs, considering approaches that are driven by theory and focusing on emerging platforms.

### Implication 3: What are the implications for future research on micro-influencers?

**Future research agenda**

The study has identified broader future research areas initially and then cluster-specific future research directions. First, the future research focus should be on strong theoretical integration. This is mainly due to the fact that current studies treat the main constructs, including authenticity, engagement, trust, and sponsorship transparency, in an isolated manner. Theorizing these as an interrelated relational process is crucial. Second, integrating diversified methodologies in future research is essential. Studies could employ experiments, longitudinal studies, a mixed-method approach, or multiple-platform comparisons. Third, the studies were skewed towards the West and Asia. This calls for exploring these concepts in different cultural and contextual settings. Future researchers should focus on how the cultural norms, values, and market conditions have an impact on authenticity and credibility. Fourth, as Instagram was the most studied single platform, future studies should analyse community features, content formats, and algorithmic visibility across emerging platforms such as YouTube, TikTok, and Twitch. Finally, the researchers should investigate under-theorized domains, including ethical disclosure practices directly linking to micro-influencers, influencer labour, creator economy, algorithmic forces, identity management, and how influencers act in socially oriented events, including sustainable communication.

The future research agenda has been curated carefully by considering the five thematic research areas, which are depicted in Table 5. This agenda covers the emerging areas and underexplored areas, providing directions to researchers, practitioners, and policymakers to carry out further explorations that intersect with trust-building, digital identity, sponsorship disclosure, value co-creation, and changing consumer-influencer relationships.

**Table 5.** Future research agenda based on research areas.

Sub- research areas	Future research agenda: Research questions
Sponsorship disclosure, influencer types and decision making: a multilevel analysis	1. How does sponsorship disclosure frequency have an impact on long-term value co-creation and the engagement of the audience? 2. How do the cultural differences affect the perception of influencer sponsorship disclosure in various regions? 3. Do nano- and micro-influencers possess the power to generate greater brand trust compared to macro- and mega-influencers for different product categories? 4. How does the credibility and trust of the influencer vary with regard to the individual characteristics in consumer decision-making stages? 5. What is the effectiveness of verified badges on consumer trust across different social media platforms? 6. To what extent do the post type and commercial intent moderate the impact of social media verified badges on consumer trust? 7. What are the effects of influencer aesthetic and video quality on response and brand evaluation across Gen Z and Millennials? 8. What is the impact of influencer type and argument quality on consumers’ neuro-physical and self-reported responses to a brand-related social media post? 9. How do consumers evaluate the source credibility of micro versus meso influencers when they are exposed to negative reviews? 10. How does text mining and natural language processing affect eWOM persuasiveness? 11. What are the core characteristics of micro-influencers that enhance brand engagement, brand love, and brand evangelism? 12. What are the micro-influencer marketing strategies utilized to be effective in promoting utilitarian versus hedonic products? 13. How are shifts in public policy, disclosure legislation affecting long-term influencer marketing effectiveness? 14. How do consumers respond to native ads when influencers use humor or emotions in their messages?
Strategic language and micro-influencer engagement,	1. How can businesses allocate budgets dynamically among micro-, meso-, and macro-influencers for maximum real-time campaign engagement? 2. How do trust and engagement get affected by different emotional tones in the influencer language used by micro versus mega influencers? 3. How does the presence of influencer-brand partnership disclosure in the influencer content affect Gen Z’s moral evaluation? 4. How can brand avoidance behavior among Gen Z be mitigated by perceived authenticity and transparency? 5. How do interaction style and follower-following ratio affect perceived authority across various industries? 6. How does the language personalization in micro-influencer content strengthen consumer-brand relationships in the long run?
Behavioural outcomes and interpersonal dynamics of micro-influencer marketing	1. How does various personal characteristics affect follower trust and brand advocacy across various types of influencers? 2. To what extent does the midset of consumers moderated the influencer marketing strategy effectiveness in tourism and wellness industry? 3. How does the parasocial relationships affect long-term consumer loyalty in micro and macro influencers? 4. How does source homophily affect engagement and perceived credibility when followers interact with influencer-generated content versus user-generated content? 5. How can influencer marketing strategies increase consumer trust and emotional bonding while facing skepticism towards sponsored content? 6. What is the impact that cultural and regional differences have on the perception of influencer credibility and effectiveness, considering the emerging markets and non-Western contexts?
Micro-influencers in identity construction and professionalization	1. To what extent does the conspicuous consumption of micro-influencers reshape traditional understandings of social status in the digital era? 2. What mechanisms enhance the perceived homophily in nano-influencers? 3. How to analyze political micro-influencers through digital forensics methods? 4. How to identify different responses of caused related communities to nano and micro-influencers in terms of loyalty, engagement and behvioral change? 5. What attitudinal drivers predict the successful professionalization of early-stage influencers? 6. What are the ways that brands and public institutions can co-create influencer content that promotes maximum public trust and engagement?
Micro-influencers as persuasive actors in niche domains.	1. How do various cultures react to micro-influencer-led public health messaging? 2. What are the long term effects of parasocial relationships across various platforms on brand loyalty? 3. What are the way to cultivated emotional solidarity and self–influencer congruence of micro-influencers? 4. What role does the algorithms play in towards content startegies? 5. How can machine learning be used to develop adaptive models for various influencer selection that focuses on brand values?

**Limitations**

Although a significant amount of time was utilized in the process of collecting and analysing the literature in this study, certain limitations exist. The study included data from a specific database, and only peer-reviewed publications were considered, excluding conference proceedings, white papers, and book chapters. This could serve as a limitation. Valuable insights that could have been derived about the rapidly evolving digital field from less formal sources can be considered overlooked. The limitation of a time frame is also significant. Articles outside the selected time frame and the ones currently under peer review have not been captured. As the study only focused on analysing articles published in English, insights from emerging economies that are published in other languages may seem underrepresented.

## Conclusion

To conclude the study, it is important to focus on the core of the study, which is micro-influencers, who were considered the social media voices, have now evolved into building connections with digital marketing, social communication, and branding. Micro-influencers were considered as the main point of the investigation as the influencer marketing arena grows into niche and sophisticated consumer segments. Researchers arrived at key conclusions based on the review. Literature built around credibility, persuasiveness, and authenticity is focused more on micro-influencers than macro and mega influencers. Micro-influencers’ ability to build parasocial relationships, have a positive impact on word-of-mouth behaviours, and ability to influence attitudes are constantly validated empirically. ^50– 52^ These findings are crucial as relational branding and consumer decision-making, heightened by emotional anchoring, are trends in marketing in the modern era. Publications are dominated by the West and Asia-Pacific, which shows an imbalance in geographic distribution and has an impact on the methodological preferences and theoretical lenses in the arena. The analysis depicts that, though the literature in the domain is growing, limited theoretical consolidation is evident. Micro and nano influencers can be used in communications that impact beyond commercial objectives. Micro-influencers are utilized in identity construction, leading to entrepreneurial pathways. Further, micro-influencers were identified as grassroots communicators in even geopolitical actors, which is leading towards unleashing the ability to influence multidimensionally.

The thematic findings identified five clusters. The clusters depict diverse interests while revealing conceptual silos. Studies focus on isolated concepts with less connection to advertising and communication mechanisms. This calls for the integration of theoretical perspectives from social proof, identity performance, digital labour, and parasocial interaction to better comprehend micro-influencer communication mechanisms. Future research directions have been identified in a broader context and cluster-specifically to address the research gaps and undertheorized areas in the arena. Overall, this review focuses on providing insights as to how the field has evolved, identifying core themes, and determining research gaps in this research area. In order to get a better understanding of the diverse roles played by micro-influencers, the development of integrated frameworks covering various levels and theoretical synthesis is suggested. Ultimately, this SLR identifies micro-influencers beyond a tool of marketing and as economic, social, and cultural actors in the virtual space. The insights from this study provide a foundation for future researchers, scholars, and practitioners to develop rigorous empirical designs and employ rich theoretical explanations to keep exploring the evolving influencer-driven communications.

## Data Availability

All the main data of this study are included as part of this article and under the references. No additional data were required. Additionally, PRISMA flow diagram, checklist, and other supplementary data are openly available in Zenodo at; https://doi.org/10.5281/zenodo.18068581. ^32^ Data are available under the terms of the Creative Commons Attribution 4.0 International license The software utilized for this study is available at: https://www.vosviewer.com/, https://posit.co/download/rstudio-desktop/, https://www.zotero.org/
