# Supplementary material for: Mapping the themes and Intellectual Structure of Micro-Influencers: A systematic Review and a Bibliometric-Analysis
Source: F1000Res. 2026 Jul 28;15:70. Originally published 2026 Jan 16. [Version 2] doi: 10.12688/f1000research.175286.2 (PMC13562925; doi:10.12688/f1000research.175286.2)
Supplement: Peer review history

# Reviewer report: Reviewer response for version 2

**DOI:** 10.5256/f1000research.206749.r516634

**Published:** 2026-09-03

**Authors:** Akhmad Edhy Aruman

**Affiliations:** 1 LSPR Instiute of Communication and Business, Jakarta, Indonesia

I have read the revised manuscript together with the responses to my comments from the authors. The suggested changes are very good and have enhanced the manuscript’s framework, its methodological explicativity and its analytical depth. My main comments have been addressed. Therefore, I have no further major comments and the manuscript in its revised version makes a relevant contribution. It can therefore be approved.

Are the rationale for, and objectives of, the Systematic Review clearly stated?

Yes

Is the statistical analysis and its interpretation appropriate?

Yes

If this is a Living Systematic Review, is the ‘living’ method appropriate and is the search schedule clearly defined and justified? (‘Living Systematic Review’ or a variation of this term should be included in the title.)

No

Are sufficient details of the methods and analysis provided to allow replication by others?

Partly

Are the conclusions drawn adequately supported by the results presented in the review?

Partly

Reviewer Expertise:

NA

I confirm that I have read this submission and believe that I have an appropriate level of expertise to confirm that it is of an acceptable scientific standard.

# Reviewer report: Reviewer response for version 2

**DOI:** 10.5256/f1000research.206749.r505867

**Published:** 2026-07-30

**Authors:** Kalpana Ambepitiya

**Affiliations:** 1 Management and Finance, General Sir John Kotelawala Defence University (Ringgold ID: 472733), Rathmalana, Western Province, Sri Lanka

The authors have satisfactorily addressed all the comments and recommendations provided during the review process. The manuscript has been revised accordingly, and the necessary improvements have been incorporated. Therefore, I am satisfied with the revised version and recommend the article for indexing.

Are the rationale for, and objectives of, the Systematic Review clearly stated?

Yes

Is the statistical analysis and its interpretation appropriate?

Partly

If this is a Living Systematic Review, is the ‘living’ method appropriate and is the search schedule clearly defined and justified? (‘Living Systematic Review’ or a variation of this term should be included in the title.)

Not applicable

Are sufficient details of the methods and analysis provided to allow replication by others?

Yes

Are the conclusions drawn adequately supported by the results presented in the review?

Partly

Reviewer Expertise:

Entrepreneurship, Marketing, Sustainable Development

I confirm that I have read this submission and believe that I have an appropriate level of expertise to confirm that it is of an acceptable scientific standard.

# Reviewer report: Reviewer response for version 1

**DOI:** 10.5256/f1000research.193256.r470676

**Published:** 2026-04-06

**Authors:** Gusti Panca

**Affiliations:** 1 Universitas Sahid, South Jakarta, Indonesia

This paper presents a systematic literature review combined with bibliometric analysis to map the development of micro-influencer research between 2020 and 2025. Using the PRISMA approach and data from Scopus (68 articles), the authors aim to identify key themes, research clusters, and future research directions. Overall, the topic is timely and relevant, especially given the rapid growth of influencer marketing and the broader creator economy.

 Regarding the clarity of the study, the rationale is generally understandable. The authors highlight the fragmentation of the literature and the need for a structured mapping of the field, which is a valid starting point. The research questions are also clearly stated. However, the justification could be strengthened from a theoretical perspective. The paper tends to position itself as a descriptive mapping exercise rather than clearly explaining what conceptual or theoretical gap it is addressing. Clarifying this would improve the overall contribution of the study.

 In terms of methodology, the use of PRISMA and the step-by-step selection process are appropriate and provide a good level of structure. The search strategy, database choice, and inclusion criteria are described, which helps readers follow the general process. However, some important details are missing for full replication. In particular, the content analysis process is not explained in sufficient depth, and there is no discussion of how coding consistency was ensured (e.g., inter-coder reliability). In addition, the criteria used to exclude certain articles such as those with “unclear methodology” are not fully transparent. Providing more detail in these areas would improve the rigor and reproducibility of the study.

 The analytical approach, which relies on bibliometric and descriptive analysis, is appropriate for mapping the literature. However, the paper refers to “meta-analysis,” which is somewhat misleading since no statistical meta-analysis (e.g., effect size estimation or heterogeneity testing) is conducted. This should be corrected either by revising the terminology or by incorporating an actual meta-analytic component.

 The conclusions are generally consistent with the results presented. The findings related to fragmented literature, dominant themes such as trust and authenticity, and geographical concentration are supported by the analysis. However, some of the broader claims especially those related to theoretical implications would benefit from a stronger and more explicit connection to the empirical findings. At times, the discussion remains descriptive rather than deeply analytical.

 With regard to the “living systematic review” aspect, the study only partially aligns with this concept. While the review is systematic and structured, it is based on a fixed dataset (2020–2025) and does not provide a clear plan for continuous updating, search frequency, or mechanisms for incorporating new studies. As such, it cannot fully be considered a living systematic review in its current form. If the authors intend it to be one, they should clearly define an update protocol.

 Overall, this paper has strong potential and addresses an important and growing research area. However, several key issues need to be addressed to strengthen its scientific contribution. These include clarifying the use of the term “meta-analysis,” improving methodological transparency (especially in the content analysis), and enhancing the theoretical integration of the findings.

**Recommendation: Major Revision**

 The manuscript is promising, but it requires improvements in conceptual clarity, methodological detail, and analytical depth before it can be considered for indexing.

**1. Are the rationale for, and objectives of, the Systematic Review clearly stated?**

**Answer: Partly**

 The overall rationale is understandable. The authors clearly point out that research on micro-influencers has grown rapidly and remains fragmented, which justifies the need for a systematic review. The focus on the 2020–2025 period is also reasonable given the post-COVID digital shift.

 That said, the paper could do a better job in terms of **theoretical positioning**. It’s not entirely clear what specific gap this study is trying to address beyond “mapping the field.” The contribution feels more descriptive than conceptual.

 The research questions are clearly stated, but they are quite broad and mainly descriptive.

**Suggestion:**

 Strengthen the theoretical framing and clarify what new insight this review brings beyond existing studies.

**2. Are sufficient details of the methods and analysis provided to allow replication by others?**

**Answer: Partly**

 The methodology is generally structured and follows PRISMA, which is a strength. The search strategy, database (Scopus), and inclusion/exclusion criteria are described, so the overall process is understandable.

 However, some important details are missing:

- The content analysis process is not explained in enough detail
- There is no mention of inter-coder reliability or how consistency was ensured
- The criteria for excluding some papers (e.g., “unclear methodology”) are not fully transparent
- The settings used in VOSviewer and Biblioshiny could be described more clearly

**Suggestion:**

 Add more detail on how the analysis was conducted, especially the coding process and validation steps, so others can replicate the study more easily.

**3. Is the statistical analysis and its interpretation appropriate?**

**Answer: Partly**

 The analytical approach (bibliometric and descriptive analysis) is appropriate for mapping the literature. However, the paper refers to “meta-analysis,” which is not actually conducted.

 There is no statistical synthesis (e.g., effect sizes, heterogeneity), so the term “meta-analysis” is misleading.

**Suggestion:**

 Either remove “meta-analysis” from the title or include a proper statistical meta-analysis.

**4. Are the conclusions drawn adequately supported by the results presented in the review?**

**Answer: Yes (with minor reservations)**

 In general, the conclusions are in line with the results. The findings about fragmentation, dominant themes (e.g., trust, authenticity), and geographical imbalance are supported by the analysis.

 However, some of the broader claims especially about theoretical gaps could be explained more clearly and linked more directly to the results.

**Suggestion:**

 Make the connection between findings and conclusions a bit tighter, especially when discussing theoretical implications.

**5. Living Systematic Review**

**Answer: Partly**

**Justification:**

 The review follows a structured and systematic approach, which reflects some characteristics of an updateable review. However, it is not explicitly designed or presented as a living systematic review. The authors use a defined time frame (2020–2025) and do not clearly outline a continuous updating process, search schedule, or protocol for incorporating new evidence over time.

**Suggestion:**

 If the authors intend this work to function as a living review, they should clearly specify an update mechanism, including the frequency of searches and criteria for incorporating new studies.

Are the rationale for, and objectives of, the Systematic Review clearly stated?

Partly

Is the statistical analysis and its interpretation appropriate?

Partly

If this is a Living Systematic Review, is the ‘living’ method appropriate and is the search schedule clearly defined and justified? (‘Living Systematic Review’ or a variation of this term should be included in the title.)

Partly

Are sufficient details of the methods and analysis provided to allow replication by others?

Partly

Are the conclusions drawn adequately supported by the results presented in the review?

Partly

Reviewer Expertise:

Influencer marketing, Systematic Literature Review, Tourism Marketing, Tourism Management

I confirm that I have read this submission and believe that I have an appropriate level of expertise to confirm that it is of an acceptable scientific standard, however I have significant reservations, as outlined above.

# Reviewer report: Reviewer response for version 1

**DOI:** 10.5256/f1000research.193256.r467584

**Published:** 2026-03-27

**Authors:** Akhmad Edhy Aruman

**Affiliations:** 1 LSPR Instiute of Communication and Business, Jakarta, Indonesia

This research can't be called a Living Systematic Review because it's missing the key parts that make a study a living review.

 The title clearly says it's a "systematic review and meta-analysis", but it doesn't mention the term "living systematic review" or anything similar, which is a basic requirement for being transparent about the study's design.

 In terms of methodology, not having this label means the authors didn't plan to use a living review approach. They should have included it if they wanted to be clear about their methods. Without it, we can't consider this study a true Living Systematic Review.

 The title and methods just don't match up with what's expected from this type of research.

 The way they did the search is more like a traditional review. They looked at a set time period, from 2020 to 2025, and used specific words to find what they were looking for, but they only did it once, in April 2025. This isn't like a living review, which needs to be updated regularly and looks at new information all the time. There's no sign that they plan to keep looking for new information or update what they found. Because of this, the review is more like a snapshot of what was known at one point in time, rather than a constantly updated collection of evidence. It doesn't have the things you'd expect to see in a living review, like repeated searches or plans to update it regularly.

 In addition, the study provides no explicit search update schedule, such as monthly, quarterly, or annual intervals, nor any justification for the frequency of updates based on the pace of new evidence generation. In a true Living Systematic Review, the update interval is typically justified by factors such as rapid publication rates, clinical urgency, or policy relevance. Although the authors acknowledge that research in this field is expanding and evolving, this observation is not translated into a structured plan for maintaining the review over time.

 From a methodological standpoint, the issue is not simply missing detail but a mismatch between the expectations of a living review design and the procedures actually implemented. A Living Systematic Review requires a clearly defined mechanism for continuous updating, transparent reporting of the update cycle, and explicit justification for adopting the living approach. None of these components are present in the current study.

 Integrated Critical Judgment

 When you look at this study using the standards for a Living Systematic Review, it doesn't quite meet the requirements. The main issue is that the method used isn't really shown to be suitable because it wasn't actually put into practice. The way they searched for information is more of a one-time thing, rather than an ongoing process, and they didn't lay out a plan for updating their findings. As a result, the study doesn't really fit the criteria for a Living Systematic Review, which means it should be seen as a regular systematic review instead of a living evidence synthesis. This is important because living systematic reviews are meant to be constantly updated, which isn't the case here.

Are the rationale for, and objectives of, the Systematic Review clearly stated?

Yes

Is the statistical analysis and its interpretation appropriate?

Yes

If this is a Living Systematic Review, is the ‘living’ method appropriate and is the search schedule clearly defined and justified? (‘Living Systematic Review’ or a variation of this term should be included in the title.)

No

Are sufficient details of the methods and analysis provided to allow replication by others?

Partly

Are the conclusions drawn adequately supported by the results presented in the review?

Partly

Reviewer Expertise:

NA

I confirm that I have read this submission and believe that I have an appropriate level of expertise to state that I do not consider it to be of an acceptable scientific standard, for reasons outlined above.

# Response

**Published:** 2026-07-18

**Authors:** LMDO Wimalasena

**Affiliations:** University of Kelaniya Faculty of Commerce and Management Studies, Kelaniya, Western Province, Sri Lanka

We thank Reviewer 2 for the detailed comments. We respectfully clarify that the manuscript was not intended, designed, or presented as a Living Systematic Review. The study is a traditional systematic literature review combined with bibliometric and content analysis, based on a fixed dataset of Scopus-indexed articles published between 2020 and 2025.

**Reviewer comment:**

*“This research can’t be called a Living Systematic Review because it’s missing the key parts that make a study a living review.”*

*“The way they did the search is more like a traditional review. They looked at a set time period, from 2020 to 2025, and used specific words to find what they were looking for, but they only did it once, in April 2025. This isn’t like a living review, which needs to be updated regularly and looks at new information all the time.”*

*“From a methodological standpoint, the issue is not simply missing detail but a mismatch between the expectations of a living review design and the procedures actually implemented. A Living Systematic Review requires a clearly defined mechanism for continuous updating, transparent reporting of the update cycle, and explicit justification for adopting the living approach. None of these components are present in the current study.”*

**Response:**

 We thank the reviewer for raising this point. We respectfully clarify that the study was not intended to be a living systematic review. We agree that a living systematic review would require an explicit updating protocol, repeated searches, a defined update schedule, and a transparent mechanism for incorporating new studies. However, our study does not claim to adopt a living review design. Instead, it is presented as a systematic review and bibliometric analysis using a fixed search period and a fixed dataset.

 To avoid any possible confusion, we have clarified the positioning of the paper as a systematic review and bibliometric analysis. The revised title now reads “Mapping the themes and intellectual structure of micro-influencers: A systematic review and a bibliometric analysis.” The methods section also clearly states that the Scopus search was conducted in April 2025 and that the study covers articles published between 2020 and 2025. Therefore, the study should be evaluated as a traditional systematic literature review and bibliometric analysis rather than as a living evidence synthesis. However, under the limitations, the fixed time frame was acknowledged by noting that articles outside the selected time frame and those under peer review were not captured.

 We respectfully believe that this comment arose from a misunderstanding of the intended review type rather than a limitation of the implemented method. Since the manuscript is not a Living Systematic Review, no update schedule or living review protocol was included.

# Reviewer report: Reviewer response for version 1

**DOI:** 10.5256/f1000research.193256.r451267

**Published:** 2026-02-14

**Authors:** Kalpana Ambepitiya

**Affiliations:** 1 Management and Finance, General Sir John Kotelawala Defence University (Ringgold ID: 472733), Rathmalana, Western Province, Sri Lanka

The article by Wimalasena and Dissanayake (2026) presents a systematic literature review and bibliometric analysis to map the intellectual structure of micro-influencer research from 2020 to 2025. The topic is timely and highly relevant, particularly given the rapid expansion of the creator economy, post-COVID digital acceleration, and the growing strategic importance of micro- and nano-influencers in marketing communication. By focusing on a recent five-year period, the authors capture contemporary shifts in digital platforms, algorithmic visibility, and evolving consumer influencer dynamics. The study is therefore positioned well within current academic and practitioner debates.

 The research questions are clearly structured and align with the objectives of bibliometric systematic reviews. The first question examines the development of micro-influencer literature, the second identifies key themes and research focuses, and the third outlines implications for future research. This logical progression enables the authors to move from descriptive mapping toward conceptual interpretation and agenda setting. The use of PRISMA enhances transparency in the selection process, reducing the dataset from 369 initial records to 68 eligible articles. The methodological procedures are clearly described, and the inclusion and exclusion criteria are systematically applied.

 A major strength of the paper lies in its methodological rigor. The integration of VOSviewer and Biblioshiny enables comprehensive visualisation of keyword co-occurrence, bibliographic coupling, authorship networks, publication trends, and thematic clustering. The identification of seven keyword clusters and five major research areas demonstrates analytical depth. The five research areas, sponsorship disclosure and decision-making, strategic language and engagement, behavioural outcomes and interpersonal dynamics, identity construction and professionalization, and niche-domain persuasion, provide a coherent structure that helps organize a previously fragmented body of literature. This clustering is one of the most valuable contributions of the study.

 The descriptive analysis further strengthens the paper by highlighting geographic concentration, citation trends, journal fragmentation, and author productivity patterns. The findings show that the literature is heavily skewed toward Western and selected high-income Asian countries, with limited representation from other regions. The authors correctly identify this as a gap affecting generalizability. Additionally, the study notes that publication growth has not always corresponded with citation impact, suggesting the need for stronger theoretical grounding in future work.

 Despite these strengths, several areas require improvement. First, the use of the term “meta-analysis” in the title is misleading. The study does not conduct a statistical meta-analysis involving effect size aggregation or quantitative synthesis of empirical findings. Instead, it performs a systematic review combined with bibliometric analysis. For academic precision and methodological clarity, the title should be revised to reflect this accurately. This is particularly important because meta-analysis implies a specific statistical approach that is not present in the paper.

 Second, while the authors repeatedly emphasize theoretical fragmentation within the micro-influencer literature, the review itself does not fully resolve this fragmentation. Although various theories such as social proof, parasocial interaction, trust transfer theory, and identity construction are mentioned, they are not synthesized into an integrated conceptual framework. The article would benefit substantially from the development of a unifying model that links sponsorship disclosure, authenticity, trust, parasocial relationships, and behavioural outcomes. Such integration would elevate the theoretical contribution beyond mapping toward theory-building.

 Third, the discussion of methodological homogeneity would benefit from stronger empirical support. The authors state that most studies are cross-sectional and that longitudinal designs are limited. However, no systematic breakdown of research designs is presented. Including a table summarizing methodological approaches such as experimental studies, surveys, qualitative research, neuroscientific experiments, and mixed methods would strengthen this claim and enhance the analytical robustness of the review.

 Additionally, although the geographic imbalance is identified, the discussion could go further in exploring why such concentration exists. Considerations such as digital infrastructure, regulatory environments, advertising disclosure laws, platform penetration rates, and cultural norms could deepen the interpretation. This would allow the paper to move from descriptive observation to structural explanation.

 From a stylistic perspective, the article would benefit from language refinement. Some sentences are lengthy and repetitive, and certain phrases are used frequently, reducing clarity. Minor grammatical inconsistencies and formatting issues (particularly in the presentation of “micro-influencers”) should be corrected to improve readability and professional presentation.

 Overall, this study makes a significant contribution by systematically mapping the evolving intellectual landscape of micro-influencer research. It provides a structured overview of thematic development, identifies research gaps, and proposes a comprehensive future research agenda. Its bibliometric mapping is rigorous and valuable for scholars entering the field. However, its theoretical integration remains moderate rather than transformative. With clearer positioning (removal of the “meta-analysis” label), stronger conceptual synthesis, and minor methodological clarifications, the paper has strong potential to become a foundational reference in micro-influencer scholarship.

Are the rationale for, and objectives of, the Systematic Review clearly stated?

Yes

Is the statistical analysis and its interpretation appropriate?

Partly

If this is a Living Systematic Review, is the ‘living’ method appropriate and is the search schedule clearly defined and justified? (‘Living Systematic Review’ or a variation of this term should be included in the title.)

Not applicable

Are sufficient details of the methods and analysis provided to allow replication by others?

Yes

Are the conclusions drawn adequately supported by the results presented in the review?

Partly

Reviewer Expertise:

Entrepreneurship, Marketing, Sustainable Development

I confirm that I have read this submission and believe that I have an appropriate level of expertise to confirm that it is of an acceptable scientific standard, however I have significant reservations, as outlined above.

# Response

**Published:** 2026-07-18

**Authors:** LMDO Wimalasena

**Affiliations:** University of Kelaniya Faculty of Commerce and Management Studies, Kelaniya, Western Province, Sri Lanka

We sincerely thank Reviewer 1 for the constructive and encouraging feedback. We appreciate the reviewer’s recognition of the timeliness of the topic, the value of the PRISMA-based systematic review, and the contribution of the bibliometric analysis and thematic clustering. We have carefully revised the manuscript to address all areas of improvement raised by the reviewer.

**Reviewer comment 1:**

*“First, the use of the term “meta-analysis” in the title is misleading. The study does not conduct a statistical meta-analysis involving effect size aggregation or quantitative synthesis of empirical findings. Instead, it performs a systematic review combined with bibliometric analysis. For academic precision and methodological clarity, the title should be revised to reflect this accurately.”*

**Response:**

 We thank the reviewer for this important clarification. We agree that the original use of the term “meta-analysis” was not methodologically appropriate because the study does not conduct statistical synthesis, effect-size aggregation, or heterogeneity testing. Therefore, we have revised the title of the manuscript from “A systematic review and meta-analysis” to “Mapping the themes and intellectual structure of micro-influencers: A systematic review and a bibliometric analysis.” **Location in revised manuscript:** Title page

**Reviewer comment 2:**

*“Second, while the authors repeatedly emphasize theoretical fragmentation within the micro-influencer literature, the review itself does not fully resolve this fragmentation. Although various theories such as social proof, parasocial interaction, trust transfer theory, and identity construction are mentioned, they are not synthesized into an integrated conceptual framework. The article would benefit substantially from the development of a unifying model that links sponsorship disclosure, authenticity, trust, parasocial relationships, and behavioural outcomes. Such integration would elevate the theoretical contribution beyond mapping toward theory-building.”*

**Response:**

 We thank the reviewer for this valuable recommendation. We agree that the paper needed to move further beyond descriptive mapping and provide stronger theoretical integration. In response, we have revised the manuscript in three locations: the introduction, the research questions section, and the discussion section. We revised the introduction to clarify the theoretical gap. The revised manuscript now explains that existing studies often examine credibility, trust, authenticity, sponsorship disclosure, engagement, parasocial interaction, and behavioural outcomes separately, creating conceptual fragmentation. This is addressed in the Introduction.

 We also revised the research questions and added an integrated conceptual framework section in the discussion. RQ3 was revised to explicitly ask how the literature on micro-influencers can be synthesised into an integrated conceptual framework. This is addressed in the research questions section.

 In the discussion section, under implication 3, Figure 16 and the accompanying paragraph present the integrated conceptual framework. This is addressed in the discussion section. The framework synthesises the literature into antecedents, mediators, moderators, contextual conditions, and outcomes of micro-influencer campaign exposure. It links constructs such as sponsorship disclosure, authenticity, trust, parasocial interaction, brand love, brand engagement, advertising literacy, digital infrastructure, and audience/brand-related outcomes. This revision strengthens the conceptual contribution and moves the review beyond descriptive mapping toward theory-informed synthesis.

**Reviewer comment 3:**

*“Third, the discussion of methodological homogeneity would benefit from stronger empirical support. The authors state that most studies are cross-sectional and that longitudinal designs are limited. However, no systematic breakdown of research designs is presented. Including a table summarizing methodological approaches such as experimental studies, surveys, qualitative research, neuroscientific experiments, and mixed methods would strengthen this claim and enhance the analytical robustness of the review.”*

**Response:**

 We thank the reviewer for this helpful suggestion. In response, we added a new section titled “Methodological profile of the studies” in the results section along with Table 4, which shows the breakdown of the research designs. This section summarises the methodological designs of the 68 included studies and shows that the literature is better described as methodologically concentrated. **Location in revised manuscript: **Results section.

**Reviewer comment 4:**

*“Additionally, although the geographic imbalance is identified, the discussion could go further in exploring why such concentration exists. Considerations such as digital infrastructure, regulatory environments, advertising disclosure laws, platform penetration rates, and cultural norms could deepen the interpretation. This would allow the paper to move from descriptive observation to structural explanation.”*

**Response:**

 We appreciate this recommendation. We agree that the original version identified geographic concentration but did not sufficiently explain why such concentration may exist. Therefore, we expanded the discussion section to provide a structural interpretation of the geographic imbalance. The revised manuscript now discusses digital infrastructure, internet penetration, platform penetration, availability of social media data, sponsorship disclosure regulations, advertising governance systems, cultural norms, English-language publication patterns, and institutional research capacity.

 This revision allows the discussion to move from descriptive observation toward structural explanation, as recommended by the reviewer. **Location in the revised manuscript: **Discussion section, under the implication 1

**Reviewer comment 5:**

*“From a stylistic perspective, the article would benefit from language refinement. Some sentences are lengthy and repetitive, and certain phrases are used frequently, reducing clarity. Minor grammatical inconsistencies and formatting issues (particularly in the presentation of “micro-influencers”) should be corrected to improve readability and professional presentation.”*

**Response:**

 Thank you for this observation. We reviewed the manuscript for language clarity, repetition, and consistency. Several sentences were shortened or revised for readability. We also improved consistency in the use of key terms. **Location in revised manuscript: **throughout the revised manuscript
